# Globally Abundant “*Candidatus* Udaeobacter” Benefits from Release of Antibiotics in Soil and Potentially Performs Trace Gas Scavenging

**DOI:** 10.1128/mSphere.00186-20

**Published:** 2020-07-08

**Authors:** Inka M. Willms, Anina Y. Rudolph, Isabell Göschel, Simon H. Bolz, Dominik Schneider, Caterina Penone, Anja Poehlein, Ingo Schöning, Heiko Nacke

**Affiliations:** a Department of Genomic and Applied Microbiology and Göttingen Genomics Laboratory, Institute of Microbiology and Genetics, Georg-August University of Göttingen, Göttingen, Germany; b Institute of Plant Sciences, University of Bern, Bern, Switzerland; c Max Planck Institute for Biogeochemistry, Jena, Germany; University of Illinois at Urbana-Champaign

**Keywords:** *Candidatus* Udaeobacter, soil, antibiotics, antibiotic resistance, *Verrucomicrobia*

## Abstract

*Verrucomicrobia* affiliated with “*Candidatus* Udaeobacter” belong to the most abundant soil bacteria worldwide. Although the synthesis of antibiotics presumably evolved in soil, and environmental pollution with antimicrobials increases, the impact of these complex molecules on “*Ca*. Udaeobacter” remains to be elucidated. In this study, we demonstrate that “*Ca.* Udaeobacter” representatives residing in grassland as well as forest soil ecosystems show multidrug resistance and even take advantage of antibiotics release. Soils treated with up to six different antibiotics exhibited a higher “*Ca.* Udaeobacter” abundance than corresponding controls after 3, 8, and 20 days of incubation. In this context, we provide evidence that “*Ca.* Udaeobacter” representatives may utilize nutrients which are released due to antibiotic-driven lysis of other soil microbes and thereby reduce energetically expensive synthesis of required biomolecules. Moreover, genomic analysis revealed the presence of genes conferring resistance to multiple classes of antibiotics and indicated that “*Ca.* Udaeobacter” representatives most likely oxidize the trace gas H_2_ to generate energy. This energy might be required for long-term persistence in terrestrial habitats, as already suggested for other dominant soil bacteria. Our study illustrates, for the first time, that globally abundant “*Ca.* Udaeobacter” benefits from release of antibiotics, which confers advantages over other soil bacteria and represents a so-far overlooked fundamental lifestyle feature of this poorly characterized verrucomicrobial genus. Furthermore, our study suggests that “*Ca.* Udaeobacter” representatives can utilize H_2_ as an alternative electron donor.

**IMPORTANCE** Soil bacteria have been investigated for more than a century, but one of the most dominant terrestrial groups on Earth, “*Candidatus* Udaeobacter,” remains elusive and largely unexplored. Its natural habitat is considered a major reservoir of antibiotics, which directly or indirectly impact phylogenetically diverse microorganisms. Here, we found that “*Ca.* Udaeobacter” representatives exhibit multidrug resistance and not only evade harmful effects of antimicrobials but even benefit from antibiotic pressure in soil. Therefore, “*Ca.* Udaeobacter” evidently affects the composition of soil resistomes worldwide and might represent a winner of rising environmental pollution with antimicrobials. In addition, our study indicates that “*Ca.* Udaeobacter” representatives utilize H_2_ and thereby contribute to global hydrogen cycling. The here-reported findings provide insights into elementary lifestyle features of “*Ca.* Udaeobacter,” potentially contributing to its successful global dissemination.

## Abstract (precis)

Soil bacteria have been investigated for more than a century, but one of the most dominant terrestrial groups on Earth, “*Candidatus* Udaeobacter,” remains elusive and largely unexplored. Its natural habitat is considered a major reservoir of antibiotics, which directly or indirectly impact phylogenetically diverse microorganisms. Here, we found that “*Ca.* Udaeobacter” representatives exhibit multidrug resistance and not only evade harmful effects of antimicrobials but even benefit from antibiotic pressure in soil. Therefore, “*Ca.* Udaeobacter” evidently affects the composition of soil resistomes worldwide and might represent a winner of rising environmental pollution with antimicrobials. In addition, our study indicates that “*Ca.* Udaeobacter” representatives utilize H_2_ and thereby contribute to global hydrogen cycling. The here-reported findings provide insights into elementary lifestyle features of “*Ca.* Udaeobacter,” potentially contributing to its successful global dissemination.

## INTRODUCTION

“*Candidatus* Udaeobacter” representatives are encountered across soil ecosystems globally (1). Nevertheless, currently, it is largely unknown how these verrucomicrobial organisms became successful terrestrial colonizers. No “*Ca.* Udaeobacter” cultures, which would allow detailed physiological analyses, are available. Moreover, *Verrucomicrobia* have been underrecognized in many studies on soil bacterial communities, since commonly used PCR primers are largely biased against their 16S rRNA genes (2). So far, the effects of antibiotics, which can be bactericidal and bacteriostatic but also beneficial, as, e.g., some bacteria can catabolize antibiotics (3, 4), have not been analyzed with respect to “*Ca.* Udaeobacter.”

A recently published metagenome-assembled genome (MAG) of “*Ca.* Udaeobacter copiosus” indicates that this phylotype exhibits auxotrophies for numerous putative vitamin and amino acid synthesis pathways (1). It is hypothesized that the essential metabolites “*Ca.* Udaeobacter copiosus” appears incapable of synthesizing are taken up from the environment, as the MAG is enriched with amino acid transporter and protease genes (1). Being dependent on extracellular metabolites in a densely colonized habitat such as soil might entail increased influx and thus vulnerability to toxic agents secreted by microorganisms competing for scarce nutrients (5). Therefore, an efficient strategy for protection against harmful substances such as antibiotics seems likely and potentially contributed to the successful global dissemination of “*Ca.* Udaeobacter.” This theory is supported by an enriched abundance of beta-lactamase genes within the phylum *Verrucomicrobia*, identified through function-based screening of soil metagenomic libraries (6).

In this study, for the first time, impacts of antibiotics on the ubiquitous soil bacterial genus “*Ca.* Udaeobacter,” a member of the *Chthoniobacterales*, were investigated. To enable robust assessment of its response to antibiotic treatment in a microcosm experiment, topsoil samples from forests and grasslands of two different geographic regions were considered. We monitored the abundance of “*Ca.* Udaeobacter” relative to that of other bacterial taxa during microcosm incubation via amplicon sequencing of 16S rRNA genes. Since primers specifically targeting this poorly characterized verrucomicrobial group are not available, we designed and evaluated oligonucleotides which we subsequently used for quantitative PCR (qPCR)-based estimation of its absolute 16S rRNA gene abundance in microcosm samples. Furthermore, a MAG of “*Ca.* Udaeobacter” was reconstructed from metagenomic sequences in order to identify antibiotic resistance genes (ARGs) and additional characteristics potentially contributing to its dominance in soil ecosystems.

We hypothesized that the abundance of “*Ca.* Udaeobacter” representatives is not reduced by an elevated concentration of the broad-spectrum antibiotic amoxicillin, as it has been indicated that beta-lactamases are enriched within *Verrucomicrobia* (6). Considering that numerous antimicrobial compounds are produced and released in soil, we further hypothesized that these globally abundant bacteria are not solely resistant to a single class of antibiotics but exhibit multidrug resistance.

## RESULTS

### Antibiotics evoke elevated “*Ca.* Udaeobacter” relative abundance.

A microcosm experiment was performed to investigate how antibiotics release affects soil bacteria representing “*Ca.* Udaeobacter.” For this experiment, initial concentrations of 77 antibiotics were determined in all forest and grassland soils used to set up the microcosms. These soils were derived from two geographic regions, Hainich-Dün and Schwäbische Alb, located in Germany (see Table S1 in the supplemental material). Except chlortetracycline (0.011 mg/kg in forest sample AEW2), which was not applied during the microcosm experiment, each of the antimicrobial compounds exhibited a concentration below the detection limit (see Table S2). Soils were treated with one antibiotic (amoxicillin), three antibiotics (amoxicillin, oxytetracycline, and sulfadiazine), or six antibiotics (amoxicillin, oxytetracycline, sulfadiazine, trimethoprim, tylosin, and ciprofloxacin) in high as well as low concentrations (corresponding controls, not treated with antibiotics, were also considered). Subsequently, we assessed the relative abundances of bacterial taxonomic groups in soil microcosms via 16S rRNA gene-based high-throughput amplicon sequencing over a period of 20 days. Prior to antibiotic treatment, strong variations of “*Ca.* Udaeobacter” relative abundances between the microcosm samples were determined. For example, the relative abundance of “*Ca.* Udaeobacter” accounted for approximately 15% in beech forest soil from the Schwäbische Alb (sample AEW7), whereas only approximately 3% of the bacterial community in a grassland soil from the Hainich-Dün region (sample HEG7) represented “*Ca.* Udaeobacter” (see Fig. S1).

10.1128/mSphere.00186-20.1 FIG S1 Relative abundance of “*Ca.* Udaeobacter” and other bacterial groups for all soil microcosms. Untreated control samples (C) as well as samples treated with antibiotics in high or low concentrations (T) were analyzed. Others, bacterial groups accounting for <2% relative abundance. Results are presented as mean from replicates. With respect to all of the four forest soil samples, treatment with one antibiotic, three antibiotics, and six antibiotics in high as well as low concentrations was performed. Four of the seven grassland soil samples were also subjected to this antibiotic treatment procedure. In addition, the remaining three grassland soil samples (AEG16, AEG21, and HEG21) were treated with three antibiotics and six antibiotics in high as well as low concentrations to further verify the effect of antibiotics release on “*Ca.* Udaeobacter.” Download FIG S1, PDF file, 0.8 MB. Copyright © 2020 Willms et al. 2020 Willms et al. This content is distributed under the terms of the Creative Commons Attribution 4.0 International license.

10.1128/mSphere.00186-20.3 TABLE S1 Description of plot characteristics and properties of the soils used to set up the microcosms and for cell extraction. Pasture, grassland with grazing livestock; meadow, grassland without grazing livestock. AEW3 was not used for the setup of the microcosms but for cell extraction. HAI, exploratory Hainich-Dün; ALB, exploratory Schwäbische Alb; LUI, land use intensity index; SMI, silvicultural management index; % water, gravimetric water content; C:N ratio, organic carbon divided by total N. Download Table S1, PDF file, 0.1 MB. Copyright © 2020 Willms et al. 2020 Willms et al. This content is distributed under the terms of the Creative Commons Attribution 4.0 International license.

10.1128/mSphere.00186-20.4 TABLE S2 List of antibiotics that have been chromatographically analyzed for residues in all grassland and forest soils used for the microcosm experiment. Only soil samples exhibiting antibiotic concentrations above the detection limits are listed. Download Table S2, PDF file, 0.1 MB. Copyright © 2020 Willms et al. 2020 Willms et al. This content is distributed under the terms of the Creative Commons Attribution 4.0 International license.

**FIG 1:**
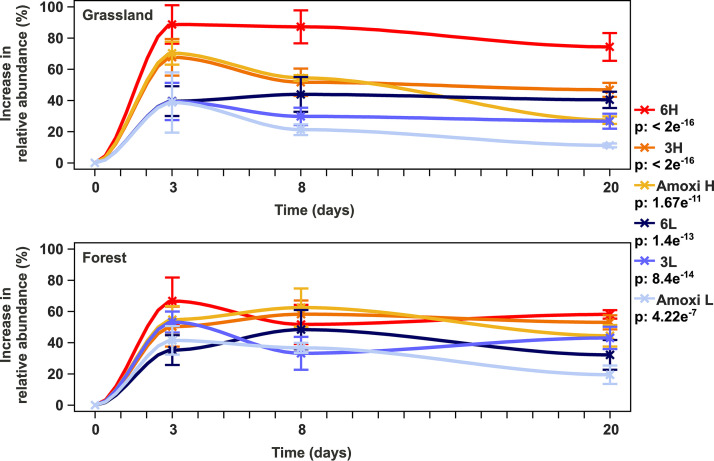
Increase in relative abundance of “*Ca.* Udaeobacter” upon antibiotic treatment across grassland and forest soil microcosms. To determine the increase in “*Ca.* Udaeobacter” relative abundance, samples treated with antibiotics were compared with corresponding controls. The increase in abundance relative to these controls is depicted. The *P* values with respect to the increase in relative 16S rRNA gene abundance in grassland and forest soil microcosms in response to different antibiotic treatments over the whole course of the experiment are indicated in the key below the respective treatment designation. Precise information about the applied linear mixed model formulas and respective quality parameters are provided in Table S3 in the supplemental material. Standard errors are indicated by vertical bars. Amoxi L, treatment with amoxicillin in low concentration; 3L, treatment with three antibiotics in low concentrations; 6L, treatment with six antibiotics in low concentrations; Amoxi H, treatment with amoxicillin in high concentration; 3H, treatment with three antibiotics in high concentrations; 6H, treatment with six antibiotics in high concentrations. Bar plots depicting abundances of “*Ca.* Udaeobacter” relative to other bacterial groups with respect to the single samples analyzed in this study are presented in Fig. S1.

Strikingly, grassland as well as forest soil microcosms treated with antibiotics exhibited significantly higher relative abundances of “*Ca.* Udaeobacter” than corresponding controls (*P* value < 2 e^−16^) (Fig. 1 and S1). This pattern was detected when a single antibiotic was added but also when three or six antibiotics were applied in both high and low concentrations (see Table S3). After 3 days of incubation, the treatment with six antibiotics in high concentrations led to the most pronounced rise of “*Ca.* Udaeobacter” relative abundance (∼50% to 100%). Furthermore, the treatments with one antibiotic and three antibiotics in high concentrations evoked similar increases in “*Ca.* Udaeobacter” relative abundance after 3 days of incubation (∼50% to 80%) (Fig. 1). With increasing days of incubation, we determined a statistically significant reduction of the antibiotic treatment effect on “*Ca.* Udaeobacter” relative abundance (*P* value < 2 e^−16^).

10.1128/mSphere.00186-20.5 TABLE S3 Model formulas and results from linear mixed model regression analysis. The relative abundance of “*Ca.* Udaeobacter” ASV 6 upon antibiotic treatment and the rise in absolute 16S rRNA gene abundance per nanogram DNA upon antibiotics release after 3 days of incubation, assessed via qPCR, were analyzed. p, *P* value; treat., antibiotic treatment; Df, degrees of freedom; Est., estimates; N, sample size. Download Table S3, PDF file, 0.1 MB. Copyright © 2020 Willms et al. 2020 Willms et al. This content is distributed under the terms of the Creative Commons Attribution 4.0 International license.

### First primer pair for targeted detection of “*Ca.* Udaeobacter.”

To verify if the abundance of “*Ca.* Udaeobacter” remains stable or even rises upon antibiotic treatment, we designed and evaluated a primer pair (UDBAC_F/UDBAC_R) for targeted detection of this verrucomicrobial group. An *in silico* analysis indicated that the primer pair covers approximately 97.14% of the “*Ca.* Udaeobacter” 16S rRNA gene sequences deposited in the SILVA SSU database (release 132). Within the evaluation process, the primer pair was used to generate amplicons based on DNA extracted from soils used to prepare the microcosms, as well as corresponding samples, incubated for 3 days after treatment with six antibiotics in high concentrations. These amplicons were subsequently subjected to high-throughput sequencing, and as expected, the generated data revealed a high preference of the UDBAC primers for “*Ca.* Udaeobacter.” Its relative abundance accounted for 99.0% ± 0.4% and 98.9% ± 0.4% of the amplicon sequences derived from untreated and treated forest soils, respectively, of both geographic regions (see Fig. S2). Furthermore, with respect to Schwäbische Alb grassland soils, 96.2% ± 3% (untreated soils) and 95.6% ± 3.9% (treated soils) of the generated amplicons represented “*Ca.* Udaeobacter” (Fig. S2). The major fraction of amplicon sequences, derived from Hainich-Dün grassland soils (untreated soil, 79.4% ± 1%; treated soil, 84.8% ± 0.9%), was also assigned to “*Ca.* Udaeobacter” (Fig. S2), but mainly due to a higher proportion of detected uncultured *Verrucomicrobiaceae*, its relative abundance was lower in Hainich-Dün grassland soils than in the other considered soils. Based on this analysis, we utilized the UDBAC primers for targeted detection and quantification of “*Ca.* Udaeobacter” 16S rRNA genes in microcosm samples.

10.1128/mSphere.00186-20.2 FIG S2 Relative 16S rRNA gene abundance of bacterial groups detected with the UDBAC primer pair designed in this study. (A) Grassland and forest soils used for microcosm preparation were considered. (B) In addition, samples incubated for three days upon treatment with six different antibiotics in high concentrations were considered. Others, bacterial groups showing less than 1% relative abundance. Download FIG S2, TIF file, 0.6 MB. Copyright © 2020 Willms et al. 2020 Willms et al. This content is distributed under the terms of the Creative Commons Attribution 4.0 International license.

### “*Ca.* Udaeobacter” benefits from antibiotics release in soil.

**FIG 2:**
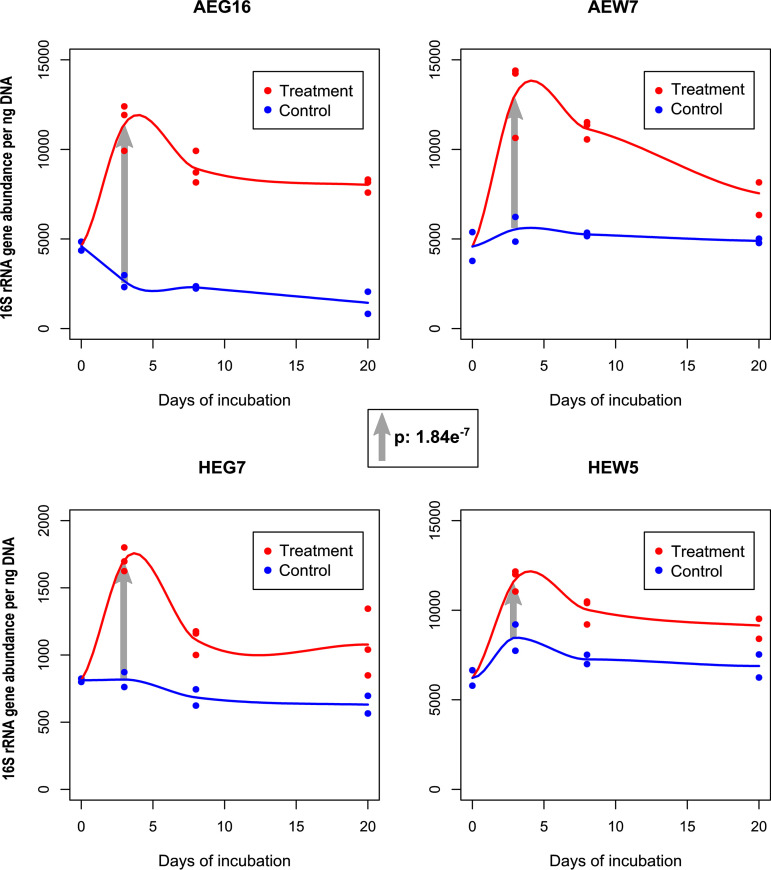
UDBAC primer pair-based determination of 16S rRNA gene abundance via qPCR. The 16S rRNA genes were quantified with respect to a grassland soil from each geographic region (samples AEG16 and HEG7) as well as a forest soil from each geographic region (samples AEW7 and HEW5). Untreated control samples (control) and samples treated with six different antibiotics in high concentrations (treatment) were considered. The *P* value with respect to the general absolute 16S rRNA gene increase in all analyzed samples after 3 days of incubation in response to antibiotic treatment (gray arrows), as calculated by a mixed linear model, is depicted in the middle. Precise information about the respective model formula and further quality parameters are provided in Table S3.

The increase of “*Ca.* Udaeobacter” abundance upon antibiotic treatment, as assessed by amplicon sequencing-based analysis, was verified via qPCR in combination with the UDBAC primers described above. This verification revealed a statistically significant rise (*P* value of 1.84 e^−7^) of 16S rRNA gene abundance in forest and grassland soil microcosms incubated for 3 days upon treatment with six different antibiotics in high concentrations (Fig. 2; Table S3). Since we found during our primer evaluation that mainly “*Ca.* Udaeobacter” is covered by the UDBAC primers in these microcosms, the rise of 16S rRNA gene abundance is, to a high degree, evoked by this verrucomicrobial group. Furthermore, similarly to the amplicon sequencing-based analysis, the qPCR data also showed that the effect of antibiotic treatment on “*Ca.* Udaeobacter” abundance decreased in the course of the microcosm experiment.

In summary, we proved that our target soil bacterial group takes advantage of antibiotics release, since its 16S rRNA gene abundance was significantly higher in treated than in control microcosms with respect to the amplicon sequencing as well as the qPCR-based data.

### “*Ca.* Udaeobacter” sp. MAG harbors several antibiotic resistance genes.

Soil-derived shotgun metagenomic sequencing data, including Oxford Nanopore and Illumina MiSeq reads, were generated to assemble a MAG of a “*Ca.* Udaeobacter” representative. In this context, we selected a forest soil sample (AEW3), extracted cells from the soil matrix, and subsequently sequenced DNA isolated from these cells. A forest soil sample was selected, as a “*Ca.* Udaeobacter” MAG from a grassland soil has already been reported (1). Additionally, several other verrucomicrobial MAGs were recently isolated from grassland soils (7). To extend the knowledge on verrucomicrobial representatives, we focused on genomic features of a “*Ca.* Udaeobacter” species inhabiting forest soil.

**FIG 3:**
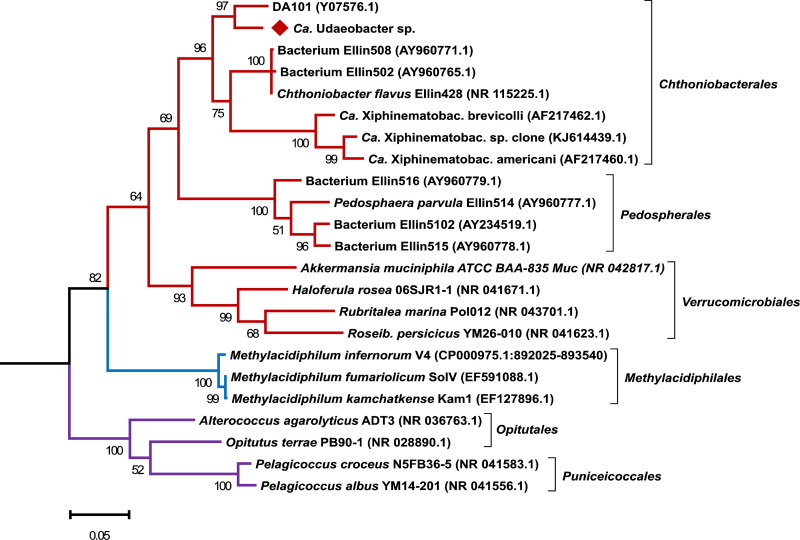
Phylogenetic analysis based on 16S rRNA gene sequences of verrucomicrobial representatives. The 16S rRNA gene sequence of the here-assembled “*Ca.* Udaeobacter” sp. MAG is highlighted with a red diamond and clusters together with the ribosomal clone DA101, affiliated with “*Ca.* Udaeobacter.” Besides the 16S rRNA gene sequence of the MAG assembled in this study, the 16S rRNA gene sequences of 22 other verrucomicrobial representatives were considered. The tree was rooted on the 16S rRNA gene sequence of Escherichia coli K-12 MG 1655 (NC_000913.3:4035531-4037072). Red, blue, and purple branches indicate the verrucomicrobial classes *Verrucomicrobiae*, *Methylacidiphilae*, and *Opitutae*, respectively. Accession numbers are given in parentheses. Bootstrap values based on 500 replicates are shown at the branching points, and the bar represents 0.05 changes per nucleotide position. All positions with less than 90% site coverage were eliminated. *Ca.* Xiphinematobac., “*Candidatus* Xiphinematobacter”; *Roseib*., *Roseibacillus*.

We assembled a 3.22-Mbp MAG comprising 145 scaffolds with an average GC content of 55.2% and an average length of 22.22 kbp (size of the longest scaffold, 92.97 kbp). It exhibited a 31-fold average Illumina MiSeq read coverage and a 64.6-fold average Oxford Nanopore read coverage. This MAG encodes 3,341 open reading frames (ORFs), one partially complete 16S rRNA gene (1,139 bp), located on a contig with a 33.7-fold average Illumina MiSeq read coverage, one 5S rRNA gene, and 38 tRNA genes. The Illumina MiSeq contig coverage indicates that the 16S rRNA gene occurs once in the genome, which is consistent with previous findings (8) and further validates its correct assignment to the genome bin. The 16S rRNA gene shows 98.83% identity to amplicon sequence variant 6 (ASV 6) (query coverage, 100%), which is the fourth-most-abundant ASV in all microcosm samples and increases significantly upon antibiotic treatment (*P* value of 1.68 e^−6^). Overall, domain-specific single-copy housekeeping gene analysis predicted 87.3% genome bin completeness with a potential contamination of 3.7%. This estimation of completeness and contamination is categorized as substantially complete with low contamination (9). The affiliation of the here-assembled genome bin to “*Ca.* Udaeobacter” was validated based on phylogenetic analysis of the nucleotide sequence of 16S rRNA genes as well as the occurrence and amino acid sequence of 120 marker genes. Regarding the 16S rRNA gene, our MAG clusters together with the ribosomal clone DA101, affiliated with “*Ca.* Udaeobacter,” and is clearly phylogenetically distinct from *Chthoniobacter* and *Xiphinematobacter*, which also represent *Chthoniobacterales* (Fig. 3).

**FIG 4:**
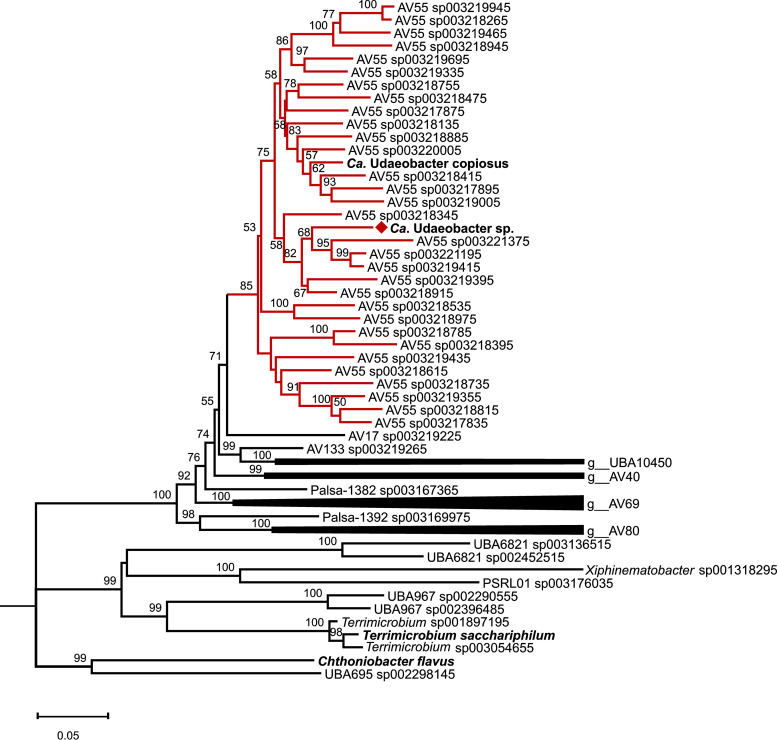
Phylogenetic tree based on the occurrence and amino acid sequence of 120 marker genes from *Chthoniobacterales*. MAGs available at GTDB as well as the “*Ca.* Udaeobacter copiosus” and the here assembled MAG were considered. The neighbor-joining tree was rooted on Escherichia coli UMN026 marker gene sequences. Red branches indicate the verrucomicrobial genus AV55 classified by GTDB, and the red diamond highlights the here-assembled “*Ca.* Udaeobacter” sp. MAG. Bootstrap values ≥50 calculated based on 500 iterations are shown at the branching points, and the bar represents 0.05 changes per amino acid position. All positions with less than 90% site coverage were eliminated.

Furthermore, the phylogenetic analysis based on the occurrence and amino acid sequence of 120 marker gene sequences of all *Chthoniobacterales* used for phylogenetic analysis (see Table S4) assigned our MAG together with “*Ca.* Udaeobacter copiosus” to the Genome Taxonomy Database (GTDB) genus AV55 (Fig. 4). As the 16S rRNA gene of our MAG is a representative of the genus “*Ca.* Udaeobacter” (Fig. 3) and “*Ca.* Udaeobacter copiosus” also clusters in the GTDB genus AV55 (Fig. 4), AV55 most likely represents the genus “*Ca.* Udaeobacter.” The closest relative of our MAG is, based on FastANI analysis, AV55 sp003218915.1, with an average nucleotide identity (ANI) of 90% over 74.6% of the genome. These values are below the ANI species threshold (10); therefore, the here-assembled MAG represents a novel species within “*Ca.* Udaeobacter,” which we designate “*Ca.* Udaeobacter” sp. in the manuscript.

10.1128/mSphere.00186-20.6 TABLE S4 Attributes of all *Chthoniobacterales* MAGs used to construct the neighbor-joining tree. The yellow highlighted MAGs cluster together with the here-assembled MAG of “*Ca.* Udaeobacter” sp. Compl, completeness estimated by Checkm; Cont, contamination estimated by Checkm; GC, GC content. Download Table S4, PDF file, 0.1 MB. Copyright © 2020 Willms et al. 2020 Willms et al. This content is distributed under the terms of the Creative Commons Attribution 4.0 International license.

**FIG 5:**
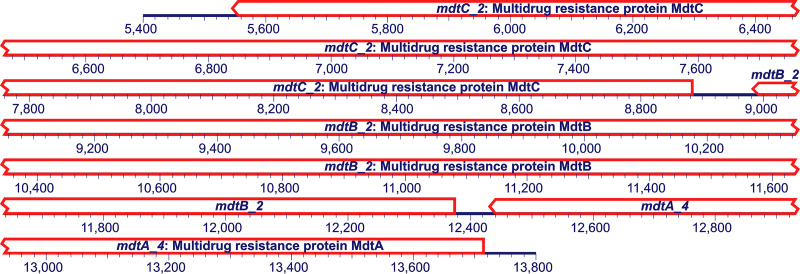
Genes potentially encoding a complete mdtABC multidrug efflux pump carried by contig 74 of the here reported “*Ca.* Udaeobacter” sp. MAG. An excerpt of the contig from 5,400 to 13,800 bp is depicted (total contig length, 16,800 bp). A detailed overview of ARGs harbored by the here-reported “*Ca.* Udaeobacter” sp. MAG can be found in Table S5.

Our MAG enabled insights into the ARG and mobile genetic element (MGE) repertoire of “*Ca.* Udaeobacter.” Based on our analysis, 55 potential ARGs and 14 MGEs were identified, which are all listed in Table S5 according to their co-occurrence on contigs. Highly abundant are genes coding for multidrug resistance mechanisms, especially subunits of resistance nodulation division (RND) MdtABC multidrug efflux systems and multidrug ABC transporters. On contig 74, a complete mdtABC efflux system is encoded, including the two RND pump genes *mdtB* and *mdtC* as well as the periplasmatic adaptor protein gene *mdtA* (Fig. 5).

10.1128/mSphere.00186-20.7 TABLE S5 Antibiotic resistance genes (ARGs) and mobile genetic elements (MGEs) encoded by the MAG of “*Ca.* Udaeobacter” sp. Download Table S5, PDF file, 0.1 MB. Copyright © 2020 Willms et al. 2020 Willms et al. This content is distributed under the terms of the Creative Commons Attribution 4.0 International license.

Furthermore, the draft genome is enriched with genes coding for macrolide efflux pump subunits, as two MacA periplasmatic adaptor, four MacB-like periplasmic core domain, and five inner membrane ATP transporter (MacB) genes are present. On contig 99, a MacB efflux pump subunit, including its periplasmic adaptor protein, and an outer membrane secretion protein HlyD are encoded. Additionally, the organism seems to be well protected from β-lactam antibiotics, due to six encoded beta-lactamases. Regarding MGEs, the MAG harbors five XerC and two XerD tyrosine recombinases as well as five insertion sequence family transposase genes. A colocation of ARGs and MGEs was not detected, as the space between the only ARG (putative chloramphenicol resistance gene) and MGE (*xerC*, encoding a tyrosine recombinase), located on one contig, is 81.675 kbp.

We also analyzed “*Ca.* Udaeobacter” sp. for the production of secondary metabolites such as antibiotics and identified a potential phosphonate synthesis cluster. From this cluster, only 4% of the genes show similarity to the known and validated thioplatensimycin biosynthetic gene cluster from Streptomyces platensis. Besides this, four gene clusters were identified which are potentially involved in terpene, arylpolyene, ladderane, and phosphonate synthesis. However, this similarity comprised only 18%, 16%, 8%, and 15% of the respective genes in the corresponding clusters, and these clusters have not been confirmed to produce a synthesis product.

### “*Ca.* Udaeobacter” sp. MAG reveals potential H_2_-based energy generation.

**FIG 6:**
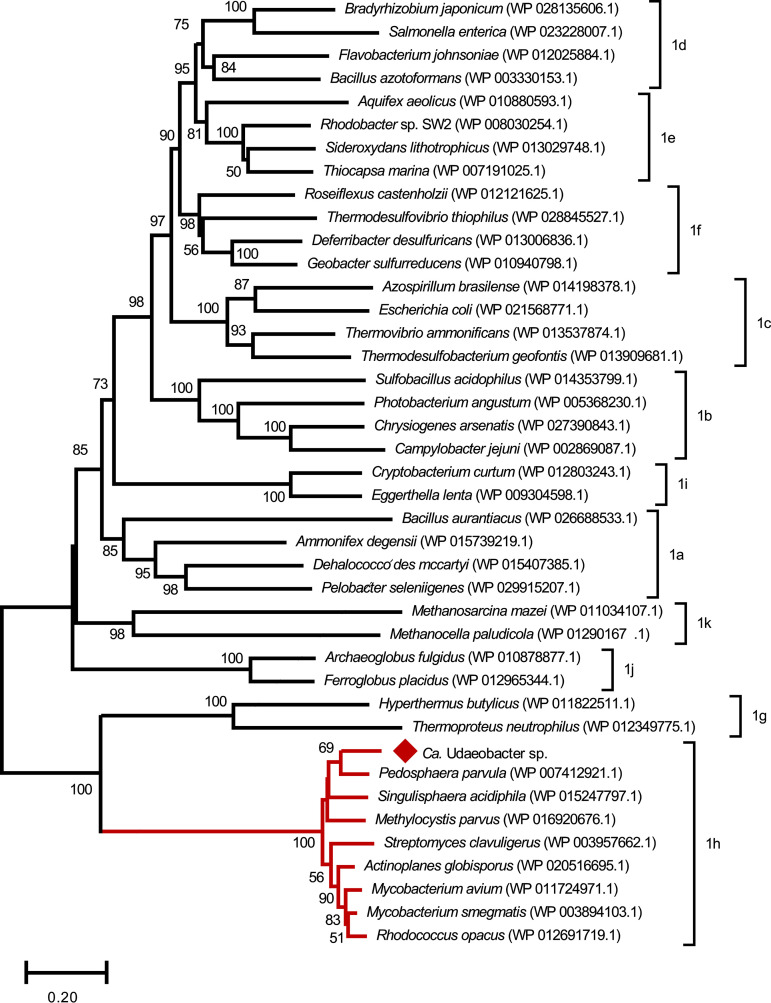
Phylogenetic tree based on the amino acid sequence of 41 large subunits of type 1 [NiFe] hydrogenases from diverse phyla, including “*Ca.* Udaeobacter” sp. (indicated with a red diamond). The red branches of the neighbor-joining tree indicate the high-affinity hydrogenase type 1h as also classified by HydDB. All other subclasses of type 1 [NiFe] hydrogenases are noted at the respective brackets. Bootstrap values of ≥50 calculated based on 500 iterations are shown at the branching points, and the bar represents 0.20 changes per amino acid position. All positions with less than 90% site coverage were eliminated.

Regarding the central metabolism encoded by the “*Ca.* Udaeobacter” sp. MAG, only one glycolysis gene is missing (the phosphoglycerate kinase gene), the pentose phosphate pathway is completely encoded and the tricarboxylic acid (TCA) cycle includes the glyoxylate bypass enzymes. Furthermore, the here-identified species harbors a small and large subunit of a high-affinity [NiFe] hydrogenase type 1h, as classified by HydDB and the here-calculated phylogenetic tree (Fig. 6). These high-affinity [NiFe] hydrogenases are known to enable the oxidation of atmospheric H_2_ to fuel the respiratory chain with electrons under nutrient-deprived conditions (11).

Regarding vitamin biosynthesis, vitamin B_12_ (cobalamin) is most likely imported from outside the cell, indicated by five encoded vitamin B_12_ import ATP-binding proteins (BtuD) and two B_12_ transporter proteins (BtuB), and further converted into its active form via the completely encoded adenosylcobalamin salvage pathway. The pathway for vitamin B_9_ (tetrahydrofolate) biosynthesis is only partially encoded, as the tetrahydrofolate reductase is missing. However, “*Ca.* Udaeobacter” is probably capable of salvaging it from 5,10-methenyltetrahydrofolate, as the respective pathway is completely encoded. Furthermore, a salvage pathway to generate thiamine (vitamin B_1_) from its degradation product and a partially complete pathway to synthesize vitamin B_6_ (four genes required for 3-amino-1-hydroxyacetone-1-phosphate synthesis from d-erythrose-4-phosphate are missing) are present in the assembled MAG.

A copper/zinc superoxide dismutase and a catalase-peroxidase gene potentially allow protection from reactive oxygen species. Furthermore, “*Ca.* Udaeobacter” sp. harbors arginine-dependent acid resistance and the biosynthesis of cadaverine from l-lysine, which protect against acidic conditions. Regarding proteinogenic amino acid biosynthesis, “*Ca.* Udaeobacter” sp. harbors complete synthesis pathways for glycine, alanine, aspartate, glutamate, glutamine, methionine, and threonine. The synthesis pathways for asparagine (1/4 enzymes missing), leucine (1/3 enzymes missing), cysteine (1/2 enzymes missing), isoleucine (3/5 enzymes missing), arginine (3/8 enzymes missing), phenylalanine (2/3 enzymes missing), tryptophan (1/12 enzymes missing), proline (2/3 enzymes missing), serine (2/3 enzymes missing), and valine (3/4 enzymes missing) are incomplete. The amino acid synthesis pathways for histidine, lysine, and tyrosine could not be identified. However, genes for two putative amino acid permeases (YdhG), which are predicted to be importers for H^+^ and an undefined amino acid, as well as for three serine/threonine exchangers (SteT), which pump out threonine into the periplasm in exchange for serine, were detected.

## DISCUSSION

The here-reported experiments clearly demonstrate that “*Ca.* Udaeobacter” representatives show multidrug resistance and even benefit from the release of antibiotics in soil. This pattern was consistently detected, though we considered samples from distinct ecosystems (beech forest, spruce forest, meadows, and pastures) of two different geographic regions. Besides the MAG reported in this study (estimated size, ∼3.67 Mbp; derived from a forest soil), another relatively small MAG (estimated size, ∼2.81 Mbp; derived from a prairie soil) of a “*Ca.* Udaeobacter” representative (“*Ca.* Udaeobacter copiosus”), which indicates multiple amino acid and vitamin auxotrophies, has been described (1, 12). Ubiquitous soil bacteria are generally known to have larger genomes in order to cope with the highly variable and rapidly changing environmental conditions in their terrestrial habitat (1, 13–15). According to Brewer et al. (1), “*Ca.* Udaeobacter copiosus” might undergo streamlining processes to reduce the metabolic expense for synthesizing costly amino acids and vitamins, which are potentially acquired from the soil environment. The here-assembled MAG of “*Ca.* Udaeobacter” sp. is missing enzymes of 13 amino acid synthesis pathways. On the other hand, 7 amino acid synthesis pathways are completely encoded. Nevertheless, it remains questionable whether the missing enzymes of the respective biosynthetic pathways imply an amino acid auxotrophy, as Price et al. (16) have shown that many missing enzymes from amino acid synthesis pathways can be filled and are predicted due to knowledge gaps in this field. However, even when all amino acid synthesis pathways are complete, the detected amino acid permeases and serine/threonine exchangers may still reduce the energy requirement for amino acid synthesis through uptake from the extracellular environment. Regarding vitamin biosynthesis, “*Ca.* Udaeobacter” sp. harbors salvage pathways for vitamin B_1_, B_12_, and B_9_ as well as transporter proteins for the import of vitamin B_12_. These indications, together with our experimental findings showing that the abundance of “*Ca.* Udaeobacter” strongly increased in microcosms treated with antibiotics, point to an efficient utilization of nutrients from soil (e.g., vitamins and amino acids) upon release of antimicrobial substances. In this context, a scavenging lifestyle of antibiotic-resistant environmental bacteria residing in nutrient-deprived habitats, proposed by Leisner et al. (5), appears possible with respect to members of “*Ca.* Udaeobacter.” Notably, the treatment of soil with one and three antibiotics in high concentrations evoked a similar increase in “*Ca.* Udaeobacter” abundance after 3 days of incubation (Fig. 1 and Fig. S1 in the supplemental material). This might be due to the fact that, in both cases, only one bactericidal antibiotic (amoxicillin) was applied, which in contrast to bacteriostatic antibiotics, kills bacteria. When a second bactericidal antibiotic was added (ciprofloxacin), a stronger increase in “*Ca.* Udaeobacter” abundance was identified after an incubation period of 3 days. With respect to treatment of soil with antibiotics in low concentrations, a similar pattern was observed after a period of 8 days. Moreover, it can be assumed that the treatment effect, inducing elevated “*Ca.* Udaeobacter” abundance, declined after prolonged incubation, as added antibiotics were degraded and therefore antimicrobial-driven cell lysis decreased over time. This observation supports our theory that “*Ca.* Udaeobacter” acquires nutrients released from cells which are killed and consequently lysed by antimicrobial compounds. Another explanation for the observed growth in response to antibiotic treatment may be the efficient exploitation of niches previously occupied by antibiotic-sensitive bacteria, without feeding on the biomass of the lysed cells. Furthermore, “*Ca.* Udaeobacter” representatives may catabolize specific antibiotics, a phenomenon which has already been reported for isolates of other soil bacteria (3, 4). To test this theory, a comparison between soil microcosms treated with selected antibiotics and control soil microcosms treated with corresponding inactive variants of the selected antibiotics might be performed in a future survey. Nevertheless, subsisting on antibiotics probably did not contribute or only marginally contributed to the observed growth of “*Ca.* Udaeobacter” upon antibiotic exposure, since supplementation with six different classes of antibiotics has been carried out and the catabolism of all here-utilized compounds is unlikely. In general, it seems plausible that the increase in “*Ca.* Udaeobacter” abundance upon release of antibiotics in soils of various ecosystems is due to a complex interaction of several advantageous attributes, such as the efficient scavenging of nutrients released through cell lysis and the concomitant exploitation of vacated environmental niches. However, to clearly elucidate the advantageous attributes of “*Ca.* Udaeobacter” that explain our observations, future studies have to be carried out.

As we verified that “*Ca.* Udaeobacter” benefits from the variety of antimicrobials applied in this study, multidrug resistance can be considered an elementary feature of representatives belonging to this genus. Accordingly, the here-assembled “*Ca.* Udaeobacter” sp. MAG encodes many multidrug efflux pumps (mostly MdtABC efflux system and ABC transporter subunits), macrolide efflux pumps (MacB ABC transporter subunits), and beta-lactam as well as other resistance genes (see Table S5). This finding is supported by Forsberg et al. (6), who reported an enrichment of beta-lactam resistance genes relative to other detected ARGs within *Verrucomicrobia* based on functional screening of agricultural and grassland soil metagenomic libraries. As, to the best of our knowledge, the antibiotic resistance repertoires of other bacterial groups have not been investigated in the forest and grassland soils considered in this study, we cannot mention specific microorganisms which are susceptible to the supplemented antibiotics. Nevertheless, due to the fact that antibiotics of up to six different classes, comprising many broad-spectrum antimicrobials, were used for treatment of the microcosm soils, it can be assumed that this treatment is harmful for several or at least a few microbes colonizing the studied samples. For instance, with respect to the synthetic broad-spectrum fluoroquinolone ciprofloxacin exhibiting a bactericidal effect against different bacteria, no resistance genes have been detected when screening 18 agricultural and grassland soil metagenomic libraries averaging 13.8 ± 8.3 (mean ± standard deviation [SD]) Gbp (6). This indicates that in some cases, the here-described antibiotic treatment even causes lysis, leading to a release of nutrients into the environment.

Antibiotic resistance and antibiotic synthesis are frequently carried out by the same bacterial species. Therefore, it was investigated whether “*Ca.* Udaeobacter” sp. also harbors biosynthesis gene clusters involved in antibiotic production. However, it seems unlikely that “*Ca.* Udaeobacter” representatives produce different antibiotics, which require large polyketide and nonribosomal peptide biosynthetic gene clusters, as we could only identify few putative genes associated with secondary metabolite biosynthesis encoded in the “*Ca.* Udaeobacter” sp. MAG. This finding is supported by potential genome streamlining and the so-far-described relatively small “*Ca.* Udaeobacter” genomes. It seems much more plausible that “*Ca.* Udaeobacter” obtains nutrients from cell lysis evoked by antibiotic producers without high energetic cost associated with the synthesis of antimicrobial compounds. In this context, the black queen hypothesis can be mentioned, which suggests that some free-living organisms “avoid” having a function in order to optimize their adaptation to the environment (17, 18). Other organisms in their close environment enable this loss of function as they publicly and continuously provide for the function, offering a (partially) stable environment (18). The mechanism underlying this particular loss of function is genome reduction through gene loss (17, 18) which likely also strongly shaped the relatively small “*Ca.* Udaeobacter” genomes.

Regarding the central metabolism, the encoded glyoxylate bypass might be advantageous with respect to bactericidal antibiotics, as it provides a means against oxidative stress. Bactericidal drugs cause a higher production of NADH through the TCA cycle, which results in a higher proportion of superoxide, formed as a consequence of respiratory chain oxidation driven by oxygen and the conversion of NADH to NAD^+^ (19, 20). Superoxides, on the other hand, cause iron leaching from iron-sulfur clusters, and iron participates in the conversion of hydrogen peroxide to hydroxyl radicals in the Fenton reaction (19). Hydroxyl radicals are extremely toxic and damage cellular proteins, lipids, and DNA, which leads to cell death and lysis (19). Bypassing the complete TCA cycle by the glyoxylate shunt reduces NADH production and causes a reduced sensitivity toward bactericidal antibiotics (21). Additionally, “*Ca.* Udaeobacter” harbors two enzymes (a copper/zinc superoxide dismutase and a catalase peroxidase) that convert superoxide to oxygen, which might also have a positive effect on the sensitivity toward bactericidal antibiotics. Nevertheless, there is an ongoing debate regarding the role of reactive oxygen species (ROS) in the activity of antibiotics (22, 23). In this context, Van Acker and Coenye (23) pointed out the importance of the experimental setup and technical aspects of the assays typically used. Another potential feature of “*Ca.* Udaeobacter” sp. is the aerobic oxidation of hydrogen indicated by a small and large subunit of high-affinity [NiFe] hydrogenase type 1h. Thus, “*Ca.* Udaeobacter” sp. is probably able to scavenge atmospheric H_2_ for energy production. This feature has been observed for *Acidobacteria* as well and was proposed as a “general mechanism for dominant soil phyla to generate the maintenance energy required for long-term survival” (24). As approximately 30% of the world’s soils are acidic (25), the acid resistance mechanisms (biosynthetic arginine decarboxylase and putative lysine decarboxylase) that were detected in the here-assembled MAG might contribute to the global dissemination of “*Ca.* Udaeobacter.” These mechanisms could increase the tolerance against acidic conditions, for example, in some forest soils, and provide an advantage over other soil bacteria.

Our study illustrates that a group of ubiquitous soil bacteria, “*Ca.* Udaeobacter,” thrives upon release of multiple classes of antibiotics. These bacteria evade growth-inhibiting and lethal effects of antimicrobials, supporting our hypotheses, and importantly, even take advantage of antibiotic pressure. Thus, steadily increasing global antibiotic consumption and an associated rising environmental pollution with antimicrobials might be advantageous for “*Ca.* Udaeobacter” representatives. Furthermore, we found that a representative of “*Ca.* Udaeobacter” harbors enzymes for hydrogen oxidation, supporting the hypothesis that trace gas scavenging might be a general mechanism of ubiquitous soil bacteria to generate energy for stable maintenance in soil. To more specifically target this verrucomicrobial group in future studies, the 16S rRNA gene-based primer pair described here will be valuable. For example, insights into the “*Ca.* Udaeobacter” phylotype composition in various soil ecosystems can be gained via sequencing of amplicons generated using the UDBAC primers. Another important step with respect to the analysis of “*Ca.* Udaeobacter” will be the cultivation of representatives belonging to this dominant group of soil bacteria. For instance, cultivates could be used to analyze potential hydrogenase activity of “*Ca.* Udaeobacter.” Moreover, the findings presented in our study indicate that application of antibiotics can contribute to a successful enrichment of “*Ca.* Udaeobacter” representatives.

## MATERIALS AND METHODS

### Sampling and soil characteristics.

Soil samples (the upper 10 cm of the mineral soil) were derived from plots of the German Biodiversity Exploratories Hainich-Dün (central Germany) and Schwäbische Alb (southwestern Germany) (26) in May 2017, as described by Solly et al. (27). Each study region covers the land use types forest and grassland. Grassland plots are 50 m by 50 m and forest plots are 100 m by 100 m in size. Detailed information on land use, dominant tree species, and fertilization for every plot is provided in Table S1 in the supplemental material. The gravimetric water content of the soil was determined by drying 10 g at 105°C for 24 h. The pH of each soil was determined as described by Solly et al. (27). For soil incubations, water contents of fresh soil samples were adjusted with deionized water to 60% water holding capacity (WHC), as the optimum water content for carbon mineralization usually falls in this range (28). The total WHC (equal to 100% WHC) was determined in the laboratory by means of disturbed soil samples. Fifteen grams of fresh soil was filled in 10-cm-high funnels which were placed in deionized water overnight until the soil was saturated with water by capillary rise. After the soil samples were drained on sand for several hours, the soil was oven dried at 105°C to a constant weight, and the soil water content at total WHC was determined. Considering the water contents of fresh soil samples, the amount of water that had to be added to the incubated samples to reach 60% WHC was then calculated.

All soils for the microcosm experiment were chromatographically analyzed for residues of 77 different antibiotics by JenaBios (Jena, Germany) (Table S2).

### Microcosm incubations.

The water content of soil derived from the selected plots was adjusted to 60% of its water holding capacity, and subsamples were frozen at −80°C in order to enable analysis of soil bacterial communities at the beginning of the microcosm experiment. Subsequently, the soil was treated with six antibiotics, three antibiotics, or one antibiotic in high and low concentrations. This procedure was performed in triplicates. The concentrations of the used antibiotics were selected based on previous microcosm experiments (29–33). Concentrations of 10 and 100 mg/kg soil of amoxicillin (Sigma-Aldrich, Steinheim, Germany), oxytetracycline dihydrate (Sigma-Aldrich), sulfadiazine (Sigma-Aldrich), trimethoprim (Sigma-Aldrich), and tylosin tartrate (Sigma-Aldrich) as well as concentrations of 5 and 50 mg/kg soil of ciprofloxacin (Sigma-Aldrich) were used for treatment with six different antibiotics. For the treatment with three antibiotics (amoxicillin, oxytetracycline dihydrate, and sulfadiazine) or one antibiotic (amoxicillin), we also used concentrations of 10 and 100 mg/kg soil. In general, many of the considered antimicrobials such as amoxicillin, ciprofloxacin, and oxytetracycline represent broad-spectrum antibiotics. Nevertheless, some bacteria can evade the harmful effects of the considered antibiotics, as for instance, amoxicillin has a broad spectrum of activity against Gram-positive bacteria but only a limited spectrum of activity with respect to Gram-negative bacteria. Therefore, and also due to differences in the antibiotic resistance repertoire of microbial taxa, it can be expected that the selected antimicrobials vary with respect to their activity toward common soil bacteria. Due to insufficient solubility in water, all antibiotics, except tylosin, were spiked onto soil in solid form. Tylosin was dissolved in sterile H_2_O prior to soil treatment. Organic solvents were avoided, as they can potentially impact soil microbial communities (30). The antibiotics were distributed in soil by vigorous mixing. Subsequently, microcosms containing 10 g of soil were prepared in 100-ml bulk flasks and incubated in the dark at 20°C. Flasks containing 10 g of soil, which was not treated with antibiotics, served as a control (set up in duplicates). The microcosms were aerated every third day to ensure oxygen supply, and the water content was ascertained to be stable via regular weighing. After 3, 8, and 20 days, samples were taken (1.25 g) and stored at −80°C for subsequent analysis.

### DNA extraction and sequencing of 16S rRNA gene amplicons.

Soil microbial community DNA was isolated from a total of 628 microcosm samples by using the DNeasy PowerSoil kit (Qiagen, Hilden, Germany) according to the manufacturer’s instructions. Subsequently, the DNA concentration was measured by employing the Qubit dsDNA Broad-Range assay kit (Thermo Fisher Scientific, Braunschweig, Germany) and a Qubit 3.0 fluorometer (Thermo Fisher Scientific) according to the manufacturer’s instructions.

The V3-V4 regions of the 16S rRNA genes were amplified by PCR using the extracted DNA from the 628 microcosm samples and the primer pair Bact-0341-b-S-17 and S-d-Bact-0785-a-A-21 (34) with modifications for Illumina MiSeq sequencing, described by Schneider et al. (35). Each PCR mixture (50 μl) contained 10 μl 5× Phusion GC buffer (Thermo Fisher Scientific), 25 ng template DNA, 0.2 μM each primer, 0.2 mM each deoxynucleoside triphosphate, 0.15 mM MgCl_2_, and 1 U Phusion High-Fidelity DNA polymerase (Thermo Fisher Scientific). PCRs were initiated at 98°C for 1 min, followed by 25 cycles of 98°C for 45 s, 66°C for 45 s, and 72°C for 30 s. The reaction ended with a final elongation step at 72°C for 5 min.

Amplicons were purified by magnetic bead purification with the automated work station Janus (PerkinElmer, Downers Grove, IL, USA) with a ratio of beads (MagSi-NGS^PREP^ Plus magnetic beads; Steinbrenner, Wiesenach, Germany) to sample of 1:1. Subsequently, indexes were added at both ends of the amplicons as described by Schneider et al. (35). Sequencing of the V3-V4 regions of the 16S rRNA genes was carried out using an Illumina MiSeq sequencer in paired-end mode and the MiSeq reagent kit v3 (600 cycles).

### Amplicon sequence data processing and statistical analysis.

The raw sequences were demultiplexed and sequencing adapters clipped by employing the data analysis software CASAVA (Illumina, San Diego, CA, USA). PEAR v.0.9.10 (36) was used for merging of paired-end reads, and sequences with a lower quality score than 20 or with unresolved bases were removed by applying the split_library_fastq.py script provided by QIIME 1.9.1 (37). Remaining forward and reverse primer sequences were removed using cutadapt 1.10 (38) with default settings. Reads of ≥380 bp were clustered into amplicon sequence variants (ASVs) (39) with the UNOISE2 algorithm (40) of USEARCH (41), which includes sequence error correction and *de novo* chimera removal. Additional chimera removal was conducted via UCHIME (42) using the SILVA SSU database (43) (release 132) as the reference. Subsequently, all quality-filtered sequences were mapped on the ASVs to determine the respective read abundance. For taxonomic classification, the ASVs were blasted against the SILVA SSU database (release 132) using the QIIME script parallel_assign_taxonomy_blast.py. Extrinsic domain ASVs, mitochondria, chloroplasts, and unclassified ASVs were removed by employing the QIIME script filter_otu_table.py. Data sets rarefied via QIIME script single_rarefaction.py to 10,000 sequences per sample were utilized for linear mixed-effect regression analysis.

The effect of antibiotic treatment on “*Ca.* Udaeobacter” was statistically analyzed using linear mixed-effect models, constructed with the R version 3.5.3 (44) and the R library lme4 (45). In this context, the logarithm of the abundance of “*Ca.* Udaeobacter” served as a response variable, the concentration of antibiotics for treatment and the days of incubation served as fixed effects, and the microcosm identification (ID) as well as the sample plot ID represented nested random effects. Furthermore, six additional models were constructed to test for significance of each treatment variation (treatment with one antibiotic, three antibiotics, and six antibiotics in high and low concentrations), where each of the six treatments served as an independent fixed effect along with the days of incubation and the nested random effects. The residuals were tested for normality and constant variance with quantile-quantile plots and residual plots using the diagnostics plots function in R. Independent variables were selected by considering collinearity, significance, and explanatory power based on *P* values, *R*^2^m and *R*^2^c, calculated with the lmerTest library (46) and the r.squaredGLMM function of the MuMIn library (47). The *P* values of the fixed and random effects were calculated with the Satterthwaite’s method and the chi-square test of the analysis of variance (ANOVA) function, respectively. All model formulas, sample sizes, corresponding *P* values, estimates, degrees of freedom, and *R*^2^ values of the conducted linear regressions are listed in Table S3.

### Design and evaluation of “*Ca.* Udaeobacter”-specific primers.

Primers for targeted detection of bacteria belonging to “*Ca.* Udaeobacter” were designed based on the 16S rRNA gene of the verrucomicrobial phylotype DA101 (48) using primer blast (49). An *in silico* PCR analysis was conducted using TestPrime 1.0 and the SILVA SSU database (34, 43) (release 132) as the reference to evaluate the specificity of designed primers (maximum of two mismatches allowed). The primer pair comprising UDBAC_F (5′-CCAGAAGAGGAAGAGACGGC-3′) and UDBAC_R (5′-GTCCTCAAGCACGGCAGTAT-3′) was used for further validation of specificity via multiplex sequencing. In this context, MiSeq overhangs described by Schneider et al. (35) were attached to each primer, and amplicons for MiSeq sequencing were produced via PCR.

Each PCR mixture (50 μl) was set up in triplicates and contained 10 μl 5× Phusion HF buffer (Thermo Fisher Scientific), 25 ng template DNA, 0.2 μM each primer, 0.2 mM each deoxynucleoside triphosphate, 1 mM MgCl_2_, and 1 U Phusion High-Fidelity DNA polymerase (Thermo Fisher Scientific). DNA extracted from all soils used for microcosm preparation and samples incubated for 3 days upon treatment with six antibiotics in high concentrations served as the templates within the PCR. The cycler program started with an initial denaturation at 98°C for 1 min followed by 25 cycles of denaturation for 10 s at 98°C, annealing at 58°C for 30 s, and elongation at 72°C for 10 s. The final elongation step was carried out at 72°C for 5 min. Generated amplicons were purified and indexed as described above. Sequencing was carried out using an Illumina MiSeq sequencer in paired-end mode and the MiSeq reagent kit v3 (600 cycles).

Bioinformatic processing of the raw data was performed as described above, except that reads shorter than 113 bp and longer than 153 bp were discarded with cutadapt 1.10.

### Quantification of “*Ca.* Udaeobacter” 16S rRNA genes.

Quantitative real-time PCR (qPCR) using primer pair UDBAC was conducted to estimate the absolute abundance of “*Ca.* Udaeobacter” 16S rRNA genes in microcosms. In a first step, a DNA fragment obtained via PCR using the UDBAC primer set was cloned into vector pCR4-TOPO (Thermo Fisher Scientific) as recommended by the manufacturer, to serve as standard for qPCR. To confirm that a partial “*Ca.* Udaeobacter” 16S rRNA gene was cloned into this vector, the insert sequence was determined by Microsynth Seqlab (Göttingen, Germany) using Sanger sequencing technology. The 16S rRNA genes of “*Ca.* Udaeobacter” representatives were quantified using an iCycler iQ5 (Bio-Rad, Hercules, CA, USA), with the QuantiNova SYBR green PCR kit (Qiagen). Each reaction mixture had a final volume of 20 μl with 10 μl of 2× QuantiNova SYBR green reverse transcriptase PCR (RT-PCR) master mix, 0.7 μM each primer, and 12.5 ng DNA template. DNA from untreated control samples and samples treated with six different antibiotics in high concentrations served as the templates. The amplification was conducted as recommended by the manufacturer with an initial activation step at 95°C for 2 min, followed by 40 cycles of denaturation at 95°C for 5 s and combined annealing and extension at 60°C for 10 s. Subsequently, melting curve analysis was conducted to ensure specific amplification.

Scatterplots depicting the 16S rRNA gene abundance of “*Ca.* Udaeobacter” per nanogram DNA in response to antibiotic treatment and sampling days were produced by employing R. In this context, a smoothed curve was generated using the loess.smooth function. The effect of the antibiotic treatment on the absolute 16S rRNA gene abundance after 3 days of incubation was statistically analyzed with a linear mixed-effect model, as described above, where the logarithm of the 16S rRNA gene copies per nanogram DNA served as a response variable, the antibiotic treatment as fixed effect, and the sample plot ID as a random effect.

### Cell extraction, sequencing, and hybrid assembly of a “*Ca.* Udaeobacter” MAG.

A frozen (−20°C) forest soil sample (AEW3) was chosen as the target for “*Ca.* Udaeobacter” genome bin assembly. Cells were extracted from the soil matrix prior to DNA extraction and sequencing. For this purpose, 100 g frozen soil was added to 200 ml morpholineethanesulfonic acid (MES) buffer (pH 5.5), supplemented with 0.24 M NH_4_Cl and 100 mg/kg amoxicillin. Subsequently, the suspension was vigorously mixed with a hand mixer for 8 min. After an incubation of 20 h at 160 rpm and 20°C, mixing was repeated, and soil particles were separated from the dissociated cells by centrifugation at 1,000 × *g* for 10 min at 4°C. Afterwards, the cells in the supernatant were pelleted at 10,000 × *g* for 30 min at 4°C and resuspended in 10 ml MES buffer. The cell suspension was pipetted onto 10 ml OptiPrep density gradient medium (Sigma-Aldrich) (1.32 g/ml iodixanol) for density gradient centrifugation at 3,000 × *g* for 90 min at 4°C. In this way, the living cells were separated from dead cell particles and other (in)organic contaminants. A thin layer above the OptiPrep layer, containing the living cells, was carefully transferred into a new vial and washed twice with 8 ml MES buffer at 10,000 × *g* for 30 min at 4°C. Finally, the cell suspension was pelleted at 10,000 × *g* for 1 h at 4°C, resuspended in 500 μl MES buffer, and stored at 4°C. High-molecular-weight DNA was isolated with the MasterPure Complete DNA & RNA purification kit (Biozym, Hessisch Oldendorf, Germany) according to the instructions in the manual. The quality of isolated DNA was initially checked by agarose gel electrophoresis and validated by using an Agilent Bioanalyzer 2100 and an Agilent DNA 12000 kit as recommended by the manufacturer (Agilent Technologies, Waldbronn, Germany). The purity of the isolated DNA was checked with a Nanodrop ND-1000 (PeqLab, Erlangen, Germany), and subsequently, the concentration was determined using the Qubit dsDNA HS assay kit (Life Technologies GmbH, Darmstadt, Germany) and a Qubit 3.0 fluorometer (Thermo Fisher Scientific). Illumina shotgun libraries were prepared using the Nextera DNA sample preparation kit and subsequently sequenced on a MiSeq system with the reagent kit v3 with 600 cycles (Illumina, San Diego, CA, USA). With respect to Oxford Nanopore sequencing, 1.5 μg DNA was used for library preparation using the Ligation Sequencing kit 1D (SQK-LSK109). Sequencing was performed for 72 h on a MinION device Mk1B using a SpotON Flow Cell R9.4.1, resulting in 16.5 million reads. Low-quality MiSeq reads and sequencing adapters were trimmed by employing trimmomatic version 0.36 in paired-end mode (50). After sequencing and trimmomatic-based quality filtering, 40.8 million MiSeq reads with an average length and paired-end insert size of 222 and 330.5 bp, respectively, were available for further processing.

Assembly was conducted by HybridSPades (51) in meta mode with automatically assessed kmer lengths. The resulting contigs were binned into MAGs with MaxBin2 (52) and checked for completeness and contamination with CheckM v1.0.12 (7). The received MAG was reassembled with HybridSPades using automatically assessed kmer lengths, and the assembly was revaluated with CheckM and QualiMap v.2.2.1 (53, 54).

Open reading frames (ORFs) were predicted and annotated using PROKKA version 1.13.4 (55). The relative abundance of an ASV, closely related to the PROKKA-annotated 16S rRNA gene in the assembled MAG, was tested for significant rise in relative abundance upon antibiotic treatment over the time course of the microcosm experiment with a linear mixed model, where the ASV abundance served as a response variable, the concentration of antibiotics for treatment and the days of incubation served as fixed effects, and the microcosm ID as well as the sample plot ID represented nested random effects (Table S3). Subsequently, the MAG was analyzed in terms of energy metabolism, amino acid auxotrophies, and environmental stress response via the PathoLogic (56) component of the Pathway Tools software (57) version 23.5 and the MetaCyc database (58). The encoded proteins were screened for annotated ARGs and MGEs. In addition, putatively novel resistance mechanisms were identified with deepARG (59) using a minimal sequence identity of 30% and a probability of >90% as thresholds. Additional MGEs were identified via DIAMOND blastx (-e 0.00001 –id 50 –subject-cover 50) against the MGE database of nanoARG (60). Furthermore, the MAG was screened for secondary metabolite biosynthesis clusters via antiSMASH 5.1 (61).

### Phylogenetic analysis.

A phylogenetic tree was constructed using MEGA X (62) based on 16S rRNA gene sequences of the here-assembled MAG and other verrucomicrobial representatives. A total of 23 nucleotide sequences were aligned with MUSCLE (63), and the tree was calculated with 500 iterations by using the maximum likelihood method and the Tamura-Nei model (64). Furthermore, the partial deletion option with a site coverage cutoff of 90% was used, and the tree was rooted with the 16S rRNA gene sequence of Escherichia coli K-12 MG1655.

The phylogenetic relation of the here-assembled MAG to other draft genomes stored in the GTDB database (65) as well as “*Ca.* Udaeobacter copiosus” was calculated based on the occurrence and amino acid sequence of 120 marker genes. The marker gene sequences identified by GTDB-tk (66) of all *Chthoniobacterales* included in the analysis (Table S4) were aligned with MUSCLE and used for calculation of a phylogenetic tree with 500 iterations by using the neighbor-joining method (67). Evolutionary distances were computed using the JTT matrix-based method (68), and the partial deletion option with a site coverage cutoff of 90% was applied. Finally, the tree was rooted based on the 16S rRNA gene sequence of E. coli UMN026 (GCA_000026325.2).

Additionally, the closest relative of our MAG was identified via FastANI based on whole-genome average nucleotide identity (69).

The phylogenetic tree of the type 1 [NiFe] hydrogenases was calculated based on 40 amino acid sequences from large [NiFe] hydrogenase subunits from diverse phyla stored at the HydDB database (70) as well as “*Ca.* Udaeobacter” sp. Each type 1 subclass is represented with two to four candidates in the tree. The amino acid sequences were aligned with MUSCLE, and the phylogenetic tree was calculated as described above, with the exception that it was left unrooted.

The large subunit of the “*Ca.* Udaeobacter” sp.-harbored [NiFe] hydrogenase was further classified by using the HydDB online hydrogenase classifier (70).

### Data availability.

The 16S rRNA gene-based amplicon sequencing data generated in this study were deposited in the Sequence Read Archive (SRA) of the NCBI under the accession number SRP226057 (BioProject accession PRJNA576637). The “*Ca.* Udaeobacter” sp. genome bin is publicly available at the NCBI under BioProject accession number PRJNA605948. This whole-genome shotgun project has been deposited at GenBank under accession number JAALOD000000000; the version described in this paper is version JAALOD010000000. Raw sequences from which the “*Ca.* Udaeobacter” sp. genome bin was assembled are available at the SRA under accession number SRP249494 (BioProject accession PRJNA605948).
