# Supplementary figures and images for: Globally Abundant “Candidatus Udaeobacter” Benefits from Release of Antibiotics in Soil and Potentially Performs Trace Gas Scavenging
Source: mSphere. 2020 Jul 8;5(4):e00186-20. doi: 10.1128/mSphere.00186-20 (PMC7343977; doi:10.1128/mSphere.00186-20)

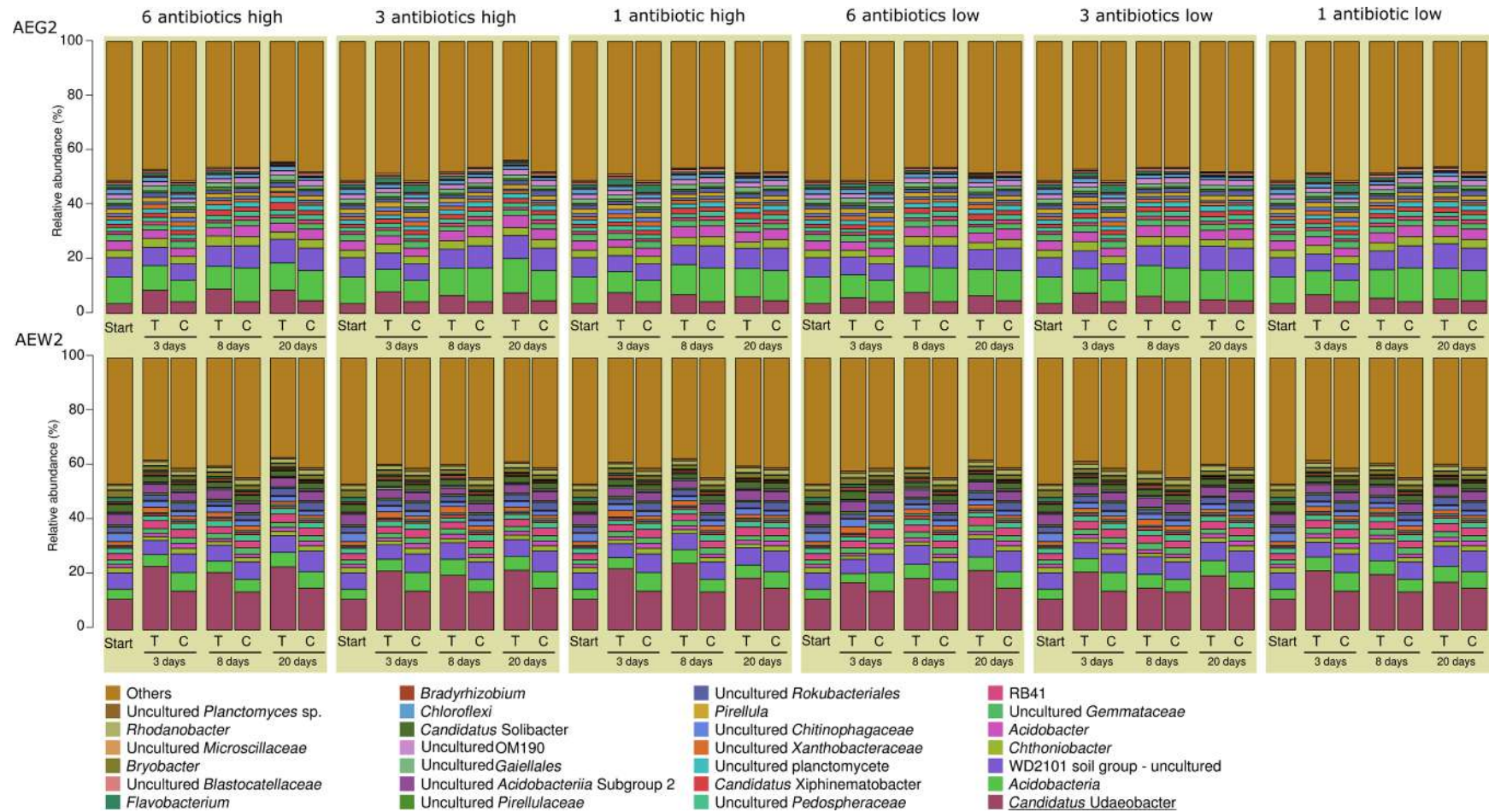

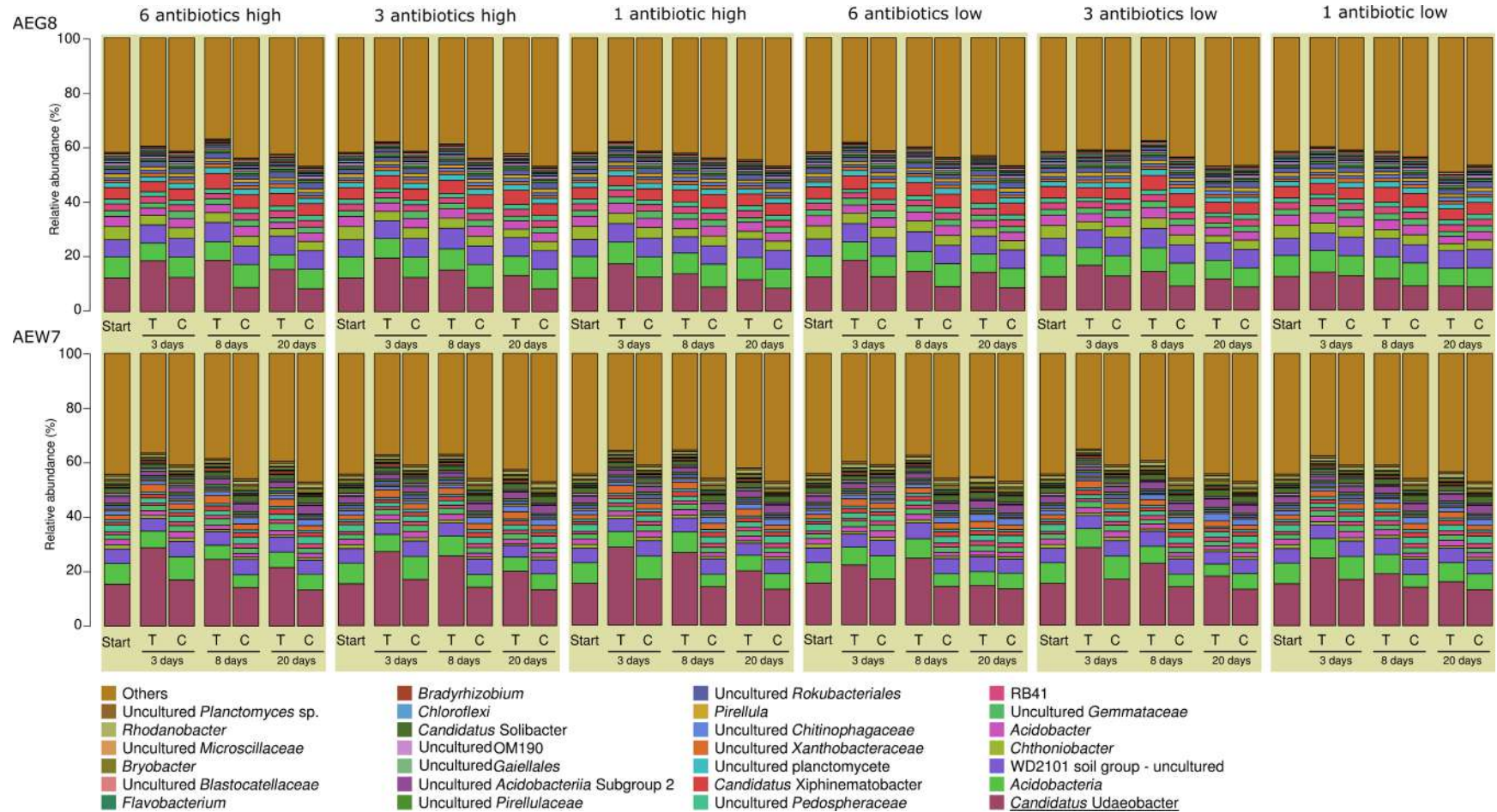

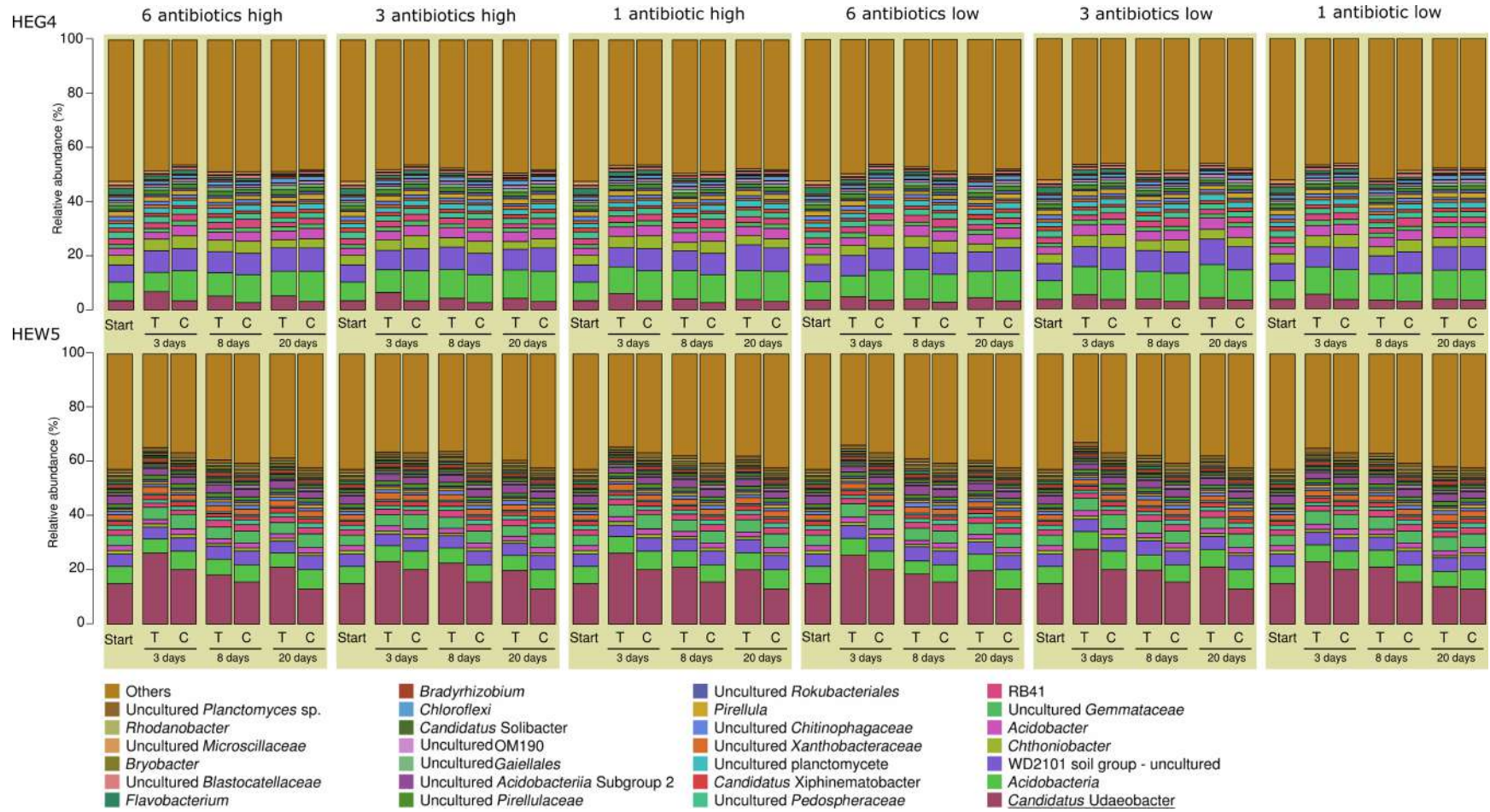

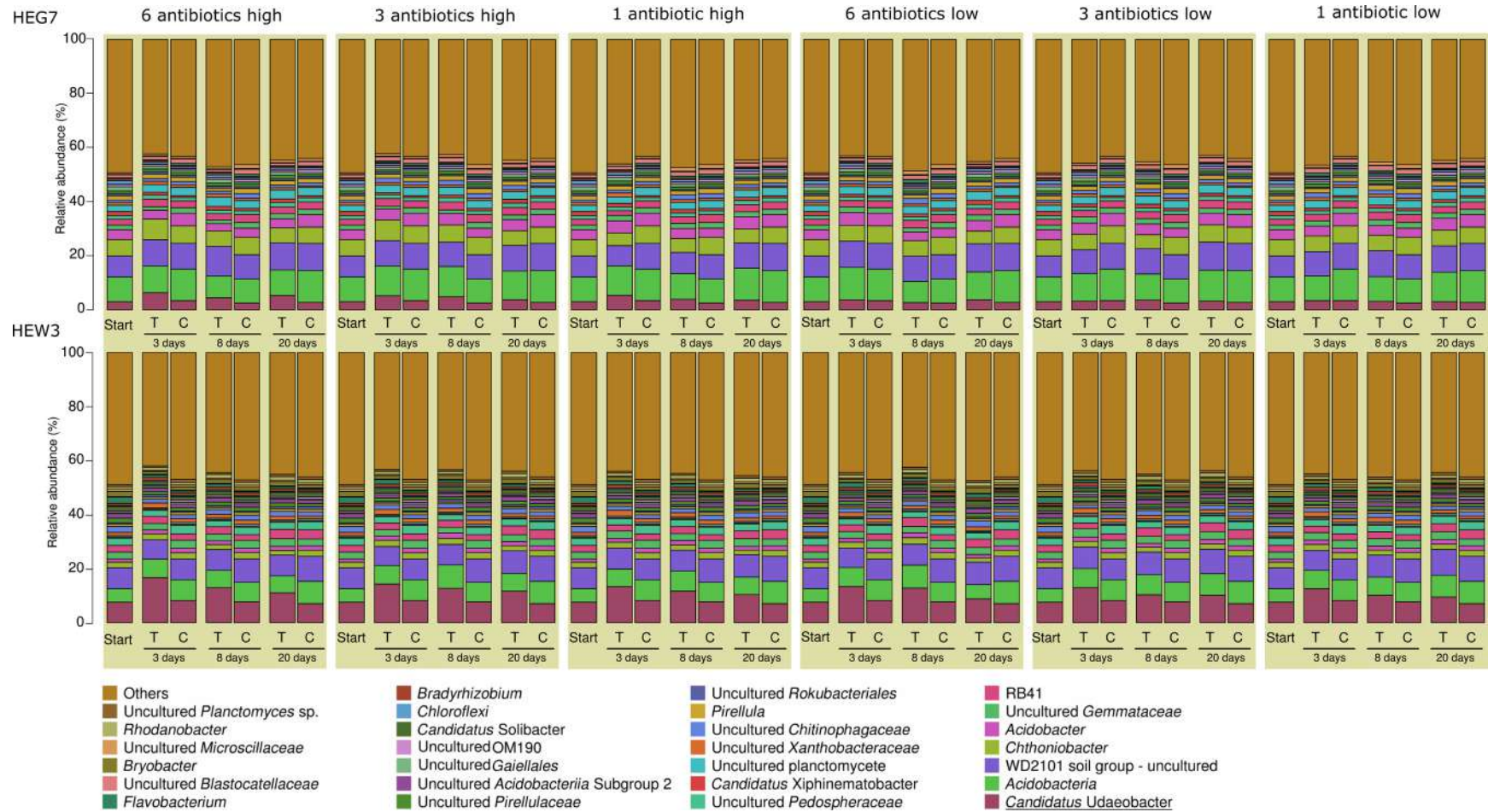

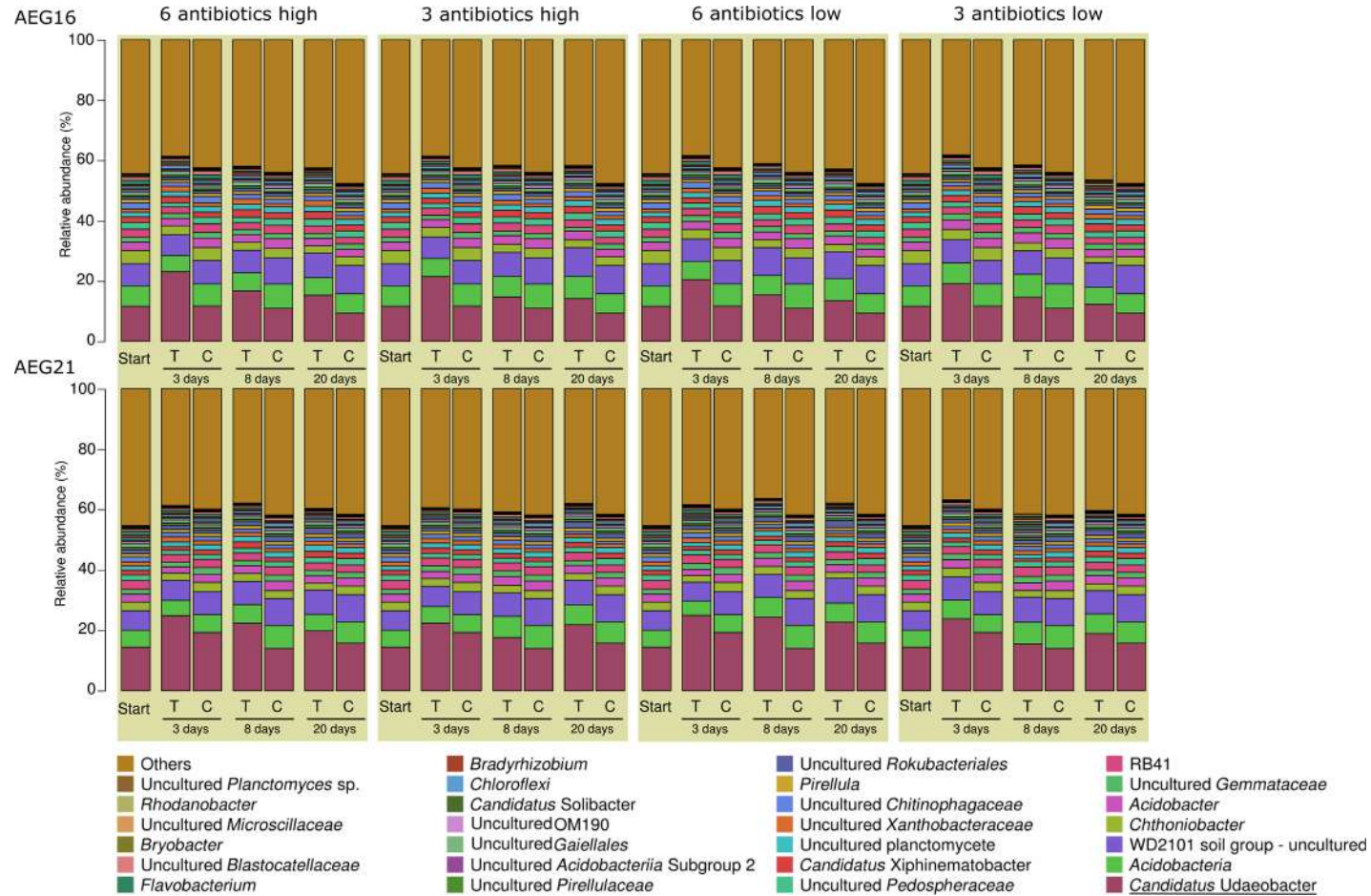

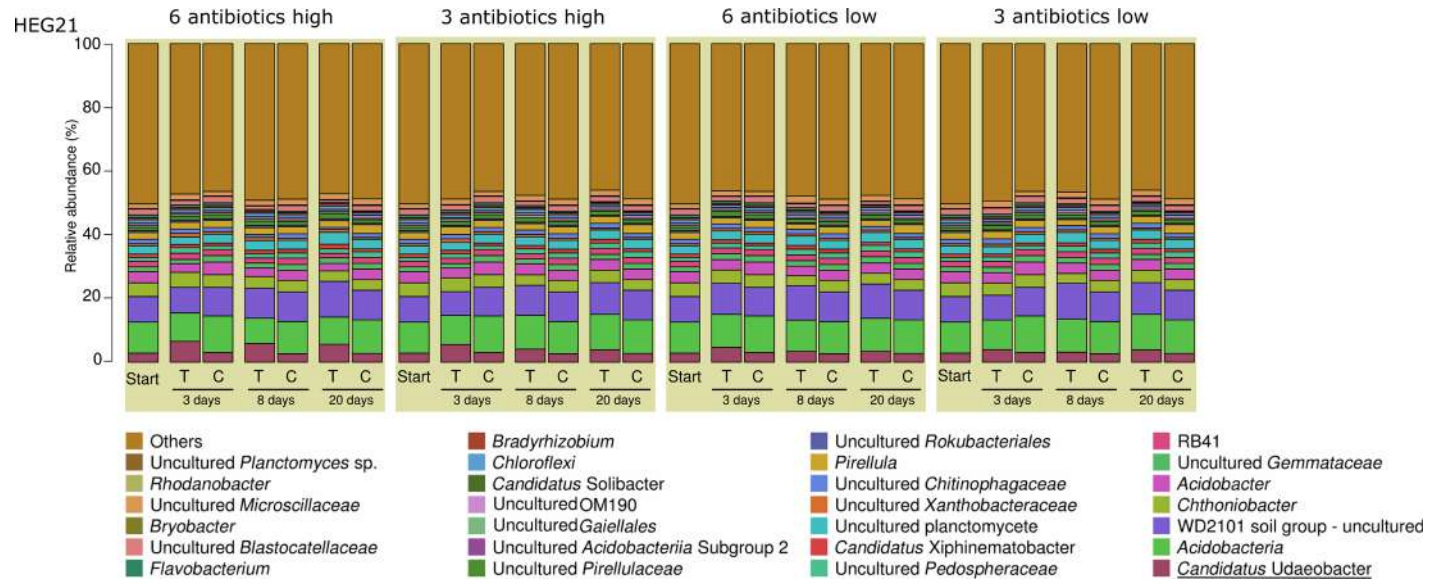

Supplement: FIG S1 [file mSphere.00186-20-sf001.pdf]

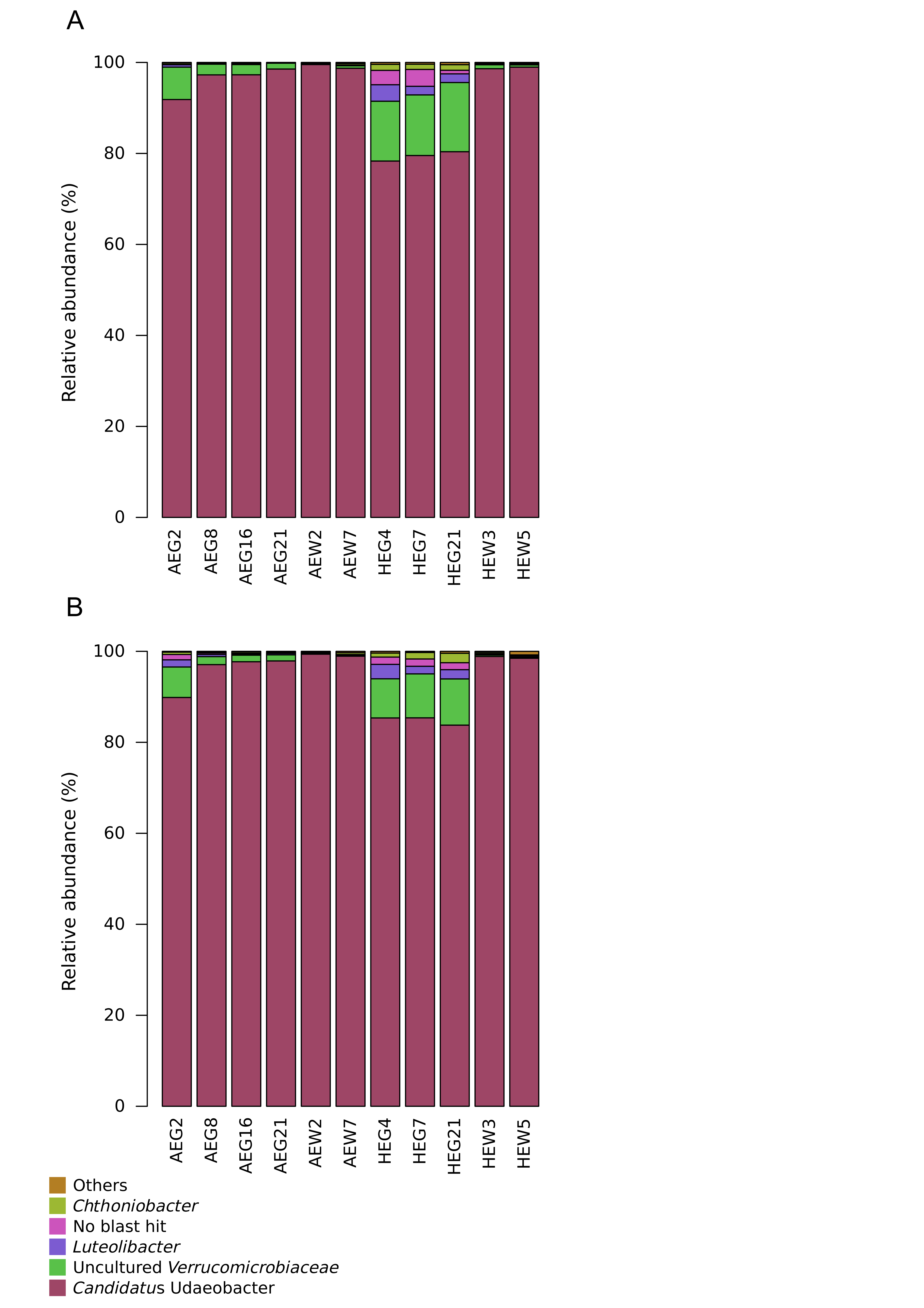

Supplement: FIG S2 [file mSphere.00186-20-sf002.tif]
