# Supplementary material for: Globally Abundant “Candidatus Udaeobacter” Benefits from Release of Antibiotics in Soil and Potentially Performs Trace Gas Scavenging
Source: mSphere. 2020 Jul 8;5(4):e00186-20. doi: 10.1128/mSphere.00186-20 (PMC7343977; doi:10.1128/mSphere.00186-20)
Supplement: TABLE S1 [file mSphere.00186-20-st001.pdf]

Plot ID	Exploratory	Plot characteristics	LUI/SMI	pH	% water	C:N ratio
HEG4	HAI	Mown, fertilized meadow	2.1	6.5	35.8	10.7
HEG7	HAI	Pasture	1.7	7	25.2	9.5
HEG21	HAI	Mown pasture	0.7	7.3	26.5	10.4
HEW3	HAI	Spruce forest	0.5	5.1	39.8	16.5
HEW5	HAI	Beech forest	0.2	5.3	52.5	13.1
AEG2	ALB	Mown, fertilized meadow	3.1	6.9	59.7	9.5
AEG8	ALB	Mown pasture	1.3	6.6	70.4	10.9
AEG16	ALB	Mown, fertilized pasture	1.7	6.0	54.7	10.2
AEG21	ALB	Mown, fertilized pasture	3.9	5.8	52.9	10.0
AEW2	ALB	Spruce forest	0.6	4.8	38.3	13.9
AEW7	ALB	Beech forest	0.2	5.0	64.9	12.9
AEW3	ALB	Spruce forest	0.5	5.6	52.7	13.6
