# Supplementary material for: Globally Abundant “Candidatus Udaeobacter” Benefits from Release of Antibiotics in Soil and Potentially Performs Trace Gas Scavenging
Source: mSphere. 2020 Jul 8;5(4):e00186-20. doi: 10.1128/mSphere.00186-20 (PMC7343977; doi:10.1128/mSphere.00186-20)
Supplement: TABLE S2 [file mSphere.00186-20-st002.pdf]

Tested antibiotics	Detection limit (mg/kg soil)	Soil sample (concentration of antibiotic)
**Sulfonamides**		
Acetylsulfadiazine	<0.001	-
Acetylsulfadimethoxine	<0.001	-
Acetylsulfamethazine	<0.001	-
Acetylsulfadoxine	<0.001	-
Acetylsulfamerazine	<0.001	-
Acetylsulfamethoxazole	<0.001	-
Acetylsulfathiazole	<0.001	-
Sulfadiazine	<0.001	-
Sulfadimethoxine	<0.001	-
Sulfamethazine	<0.001	-
Sulfadoxine	<0.001	-
Sulfamerazine	<0.001	-
Sulfamethoxazole	<0.001	-
Sulfathiazole	<0.001	-
**Penicillins**		
Amoxicillin	<0.01	-
Ampicillin	<0.01	-
Benzylpenicillin	<0.01	-
Cloxacillin	<0.01	-
Dicloxacillin	<0.01	-
Oxacillin	<0.01	-
Penethamate	<0.01	-
Penicillin V	<0.01	-
**Aminoglycosides**		
Apramycin	<0.01	-
Clindamycin	<0.01	-
Gentamycin C1a	<0.05	-
Lincomycin	<0.01	-
Neomycin	<0.01	-
Spectinomycin	<0.05	-
Streptomycin	<0.01	-
**Polypeptides**		
Bacitracin A	<0.01	-
Collistin	<0.03	-
Nafcillin	<0.01	-
Nisin	<0.01	-
**Cephalosporins**		
Cefadroxil	<0.01	-
Cefapirin	<0.01	-
Cefazolin	<0.01	-
Cefquinome	<0.01	-
Ceftiofur	<0.01	-
Cefuroxime	<0.01	-
Cephalexin	<0.01	-
**Amphenicols**		
Chloramphenicol	<0.001	-
Thiamphenicol	<0.001	-
**Tetracyclines**		
Florfenicol	<0.001	-

Tested antibiotics	Detection limit (mg/kg soil)	Soil sample (concentration of antibiotic)
Chlortetracycline	<0.01	AEW2 (0.011 mg/kg)
Demeclocycline	<0.003	-
Doxycycline	<0.01	-
Iso-Chlortetracycline	<0.01	-
Meclocycline	<0.01	-
Metacycline	<0.01	-
Oxytetracycline	<0.01	-
Tetracycline	<0.01	-
**Quinolones**		
Ciprofloxacin	<0.001	-
Danofloxacin	<0.01	-
Difloxacin	<0.01	-
Enrofloxacin	<0.01	-
Marbofloxacin	<0.01	-
Ofloxacin	<0.01	-
Sarafloxacin	<0.01	-
**Macrolides**		
Clarithromycin	<0.01	-
Erythromycin	<0.01	-
Flavomycin	<0.01	-
Ivermectin B1a	<0.05	-
Natamycin	<0.01	-
Oleandromycin	<0.01	-
Spiramycin S-I	<0.05	-
Spiramycin S-II	<0.01	-
Spiramycin S-III	<0.01	-
Tilmicosin	<0.01	-
Tylosin	<0.05	-
**Nitroimidazoles**		
Metronidazole	<0.01	-
Ronidazole	<0.01	-
**Additional antibiotics**		
Imipenem	<0.01	-
Tiamulin	<0.001	-
Trimethoprim	<0.01	-
Tylvalosin	<0.01	-
Valnemulin	<0.01	-
Virginamycin	<0.01	-
