# Supplementary material for: Globally Abundant “Candidatus Udaeobacter” Benefits from Release of Antibiotics in Soil and Potentially Performs Trace Gas Scavenging
Source: mSphere. 2020 Jul 8;5(4):e00186-20. doi: 10.1128/mSphere.00186-20 (PMC7343977; doi:10.1128/mSphere.00186-20)
Supplement: TABLE S3 [file mSphere.00186-20-st003.pdf]

Model formula	N	R^2m/R^2c	p treat.	Df treat.	Est. treat.	p day	Df day	Est. day
lmer(log(Ca. Udaeobacter)~Concentr+Days+(1|plotID/sampleID))	606	0.04/ 0.96	$< 2\ e^{-16}$	190	$6.0\ e^{-4}$	$< 2\ e^{-16}$	403	-0.01
lmer(log(Ca. Udaeobacter)~SixAntib.High+Days+(1|plotID/sampleID)	83	0.15/ 0.96	$< 2\ e^{-16}$	38	$9.8\ e^{-4}$	$1.8\ e^{-4}$	66	-0.01
lmer(log(Ca. Udaeobacter)~SixAntib.Low+Days+(1|plotID/sampleID)	79	0.06/ 0.96	$1.4\ e^{-13}$	66	0.01	$6.9\ e^{-4}$	66	-0.01
lmer(log(Ca. Udaeobacter)~ThreeAntib.High+Days+(1|plotID/sampleID)	87	0.08/ 0.95	$< 2\ e^{-16}$	74	$1.4\ e^{-3}$	$7.4\ e^{-4}$	74	-0.01
lmer(log(Ca. Udaeobacter)~ThreeAntib.Low+Days+(1|plotID/sampleID)	88	0.04/ 0.96	$8.4\ e^{-14}$	75	0.01	$9.9\ e^{-4}$	75	-0.01
lmer(log(Ca. Udaeobacter)~OneAntib.High+Days+(1|plotID/sampleID)	65	0.1/ 0.97	$1.67\ e^{-11}$	25	$4.5\ e^{-3}$	$2.5\ e^{-5}$	50	-0.01
lmer(log(Ca. Udaeobacter)~OneAntib.Low+Days+(1|plotID/sampleID)	66	0.04/ 0.96	$4.22\ e^{-7}$	53	0.02	$3.0\ e^{-5}$	52	-0.01
lmer(ASV 6~Concentr+Days+(1|plotID/sampleID))	606	0.02/0.90	$1.68\ e^{-6}$	593	0.04	$< 2\ e^{-16}$	593	-1.73
lmer(log(16S rRNA genes per ng DNA after 3 days)~Concentr+(1| plotID))	30	0.02/ 0.90	$1.84\ e^{-7}$	23	$1.6\ e^{-3}$	NA.	NA	NA
