# Supplementary material for: Globally Abundant “Candidatus Udaeobacter” Benefits from Release of Antibiotics in Soil and Potentially Performs Trace Gas Scavenging
Source: mSphere. 2020 Jul 8;5(4):e00186-20. doi: 10.1128/mSphere.00186-20 (PMC7343977; doi:10.1128/mSphere.00186-20)
Supplement: TABLE S4 [file mSphere.00186-20-st004.pdf]

GBK assembly accession	GTDB Phylogeny starting with the family	Compl.	Cont.	Size in Mbp	GC
GCA_003221405.1	f__UBA10450;g__AV69;s__AV69 sp003221405	66.6%	2.0%	1.94	55.4%
GCA_003218375.1	f__UBA10450;g__AV80;s__AV80 sp003218375	100.0%	2.7%	3.96	58.8%
GCA_003218735.1	f__UBA10450;g__AV55;s__AV55 sp003218735	72.0%	2.6%	1.49	54.5%
GCA_003218615.1	f__UBA10450;g__AV55;s__AV55 sp003218615	88.3%	0.5%	2.29	55.3%
GCA_003219225.1	f__UBA10450;g__AV17;s__AV17 sp003219225	70.5%	0.7%	2.04	58.4%
GCA_003219215.1	f__UBA10450;g__AV40;s__AV40 sp003219215	90.8%	1.7%	2.33	55.3%
GCA_003219095.1	f__UBA10450;g__AV69;s__AV69 sp003219095	71.4%	3.2%	2.38	55.1%
GCA_003244145.1	f__UBA10450;g__AV80;s__AV80 sp003244145	92.9%	5.1%	3.63	58.4%
GCF_001613545.1	f__Terrimicrobiaceae;g__Terrimicrobium;s__Terrimicrobium sacchariphilum	100.0%	2.7%	4.75	60.2%
GCA_003219465.1	f__UBA10450;g__AV55;s__AV55 sp003219465	89.1%	6.6%	4.19	55.2%
GCA_003134785.1	f__UBA10450;g__AV80;s__AV80 sp003134785	100.0%	3.4%	4.39	57.8%
GCA_003218885.1	f__UBA10450;g__AV55;s__AV55 sp003218885	88.0%	2.4%	2.30	55.4%
GCF_000173075.1	f__Chthoniobacteraceae;g__Chthoniobacter;s__Chthoniobacter flavus	97.3%	2.9%	7.85	61.1%
GCA_003221295.1	f__UBA10450;g__AV69;s__AV69 sp003221295	90.2%	3.7%	2.98	55.3%
GCA_003219175.1	f__UBA10450;g__AV69;s__AV69 sp003219175	98.0%	5.7%	3.24	55.0%
GCA_003220155.1	f__UBA10450;g__AV69;s__AV69 sp003220155	96.3%	3.0%	3.35	55.3%
GCA_003167555.1	f__UBA10450;g__AV80;s__AV80 sp003167555	100.0%	3.4%	4.20	58.5%
GCA_003219195.1	f__UBA10450;g__UBA10450;s__UBA10450 sp003219195	95.3%	2.2%	2.82	55.8%
GCA_003219925.1	f__UBA10450;g__AV69;s__AV69 sp003219925	81.2%	3.0%	2.10	55.3%
GCA_003219355.1	f__UBA10450;g__AV55;s__AV55 sp003219355	88.4%	6.4%	2.23	54.5%
GCA_003445855.1	f__UBA10450;g__UBA10450;s__UBA10450 sp003445855	78.0%	1.7%	2.10	55.9%
GCA_003219995.1	f__UBA10450;g__UBA10450;s__UBA10450 sp003219995	82.6%	2.5%	2.10	55.3%
GCA_003221375.1	f__UBA10450;g__AV55;s__AV55 sp003221375	73.9%	1.4%	2.17	54.1%
GCA_003218535.1	f__UBA10450;g__AV55;s__AV55 sp003218535	92.6%	4.4%	2.69	54.5%
GCA_003167365.1	f__UBA10450;g__Palsa-1382;s__Palsa-1382 sp003167365	100.0%	1.4%	3.13	55.2%
GCA_003219495.1	f__UBA10450;g__AV40;s__AV40 sp003219495	72.9%	1.5%	2.11	56.3%
GCA_003218305.1	f__UBA10450;g__AV80;s__AV80 sp003218305	92.9%	4.6%	3.60	59.0%
GCA_003169695.1	f__UBA10450;g__AV80;s__AV80 sp003169695	99.7%	4.7%	4.14	57.2%
GCA_001897195.1	f__Terrimicrobiaceae;g__Terrimicrobium;s__Terrimicrobium sp001897195	96.6%	3.8%	5.06	60.8%
GCA_003219125.1	f__UBA10450;g__AV69;s__AV69 sp003219125	92.3%	4.6%	3.20	55.0%
GCA_003219435.1	f__UBA10450;g__AV55;s__AV55 sp003219435	86.4%	2.4%	2.73	55.2%

GBK assembly accession	GTDB Phylogeny	Compl.	Cont.	Size in Mbp	GC
GCA_003220055.1	f__UBA10450;g__AV40;s__AV40 sp003220055	81.8%	0.2%	2.08	55.5%
GCA_003219335.1	f__UBA10450;g__AV55;s__AV55 sp003219335	83.0%	4.1%	3.71	55.0%
GCA_002290555.1	f__*Terrimicrobiaceae*;g__UBA967;s__UBA967 sp002290555	81.1%	0.2%	1.82	56.6%
GCA_003136515.1	f__UBA6821;g__UBA6821;s__UBA6821 sp003136515	85.1%	4.1%	2.36	53.2%
GCA_002298145.1	f__*Chthoniobacteraceae*;g__UBA695;s__UBA695 sp002298145	92.6%	0.7%	4.11	60.5%
GCA_003218415.1	f__UBA10450;g__AV55;s__AV55 sp003218415	83.7%	3.4%	2.05	55.3%
GCA_003219005.1	f__UBA10450;g__AV55;s__AV55 sp003219005	87.8%	1.1%	2.69	55.2%
GCA_003218975.1	f__UBA10450;g__AV55;s__AV55 sp003218975	98.0%	2.9%	3.06	55.1%
GCA_003218785.1	f__UBA10450;g__AV55;s__AV55 sp003218785	85.6%	0.7%	2.78	54.9%
GCA_003218705.1	f__UBA10450;g__AV40;s__AV40 sp003218705	76.5%	0.8%	1.70	55.4%
GCA_003218945.1	f__UBA10450;g__AV55;s__AV55 sp003218945	87.8%	3.1%	2.80	55.0%
GCA_003219265.1	f__UBA10450;g__AV133;s__AV133 sp003219265	78.4%	1.4%	2.09	56.2%
GCA_003218135.1	f__UBA10450;g__AV55;s__AV55 sp003218135	91.2%	3.7%	2.75	54.9%
GCA_003169975.1	f__UBA10450;g__Palsa-1392;s__Palsa-1392 sp003169975	100.0%	2.7%	4.47	59.9%
GCF_001318295.1	f__*Xiphinematobacteraceae*;g__*Xiphinematobacter*;s__*Xiphinematobacter* sp001318295	89.9%	0.0%	0.92	47.7%
GCA_003244125.1	f__UBA10450;g__AV69;s__AV69 sp003244125	66.0%	2.3%	2.50	58.7%
GCA_003217965.1	f__UBA10450;g__AV69;s__AV69 sp003217965	90.3%	3.7%	2.64	55.2%
GCA_003218475.1	f__UBA10450;g__AV55;s__AV55 sp003218475	94.7%	3.6%	3.16	54.8%
GCA_003134765.1	f__UBA10450;g__AV80;s__AV80 sp003134765	96.0%	2.7%	4.05	58.7%
GCA_003221195.1	f__UBA10450;g__AV55;s__AV55 sp003221195	89.6%	4.1%	3.10	54.3%
GCA_003218345.1	f__UBA10450;g__AV55;s__AV55 sp003218345	82.5%	3.2%	2.39	54.5%
GCA_003218915.1	f__UBA10450;g__AV55;s__AV55 sp003218915	88.7%	3.3%	2.91	54.6%
GCA_003220075.1	f__UBA10450;g__AV69;s__AV69 sp003220075	87.4%	4.5%	2.79	55.2%
GCA_003176035.1	f__*Xiphinematobacteraceae*;g__PSRL01;s__PSRL01 sp003176035	70.4%	3.4%	1.92	48.5%
GCF_003054655.1	f__*Terrimicrobiaceae*;g__*Terrimicrobium*;s__*Terrimicrobium* sp003054655	100.0%	2.0%	4.75	60.7%
GCA_003218265.1	f__UBA10450;g__AV55;s__AV55 sp003218265	66.6%	2.2%	2.25	55.0%
GCA_003218815.1	f__UBA10450;g__AV55;s__AV55 sp003218815	77.1%	2.3%	1.85	54.6%
GCA_003218395.1	f__UBA10450;g__AV55;s__AV55 sp003218395	90.7%	4.4%	2.95	54.9%
GCA_003219695.1	f__UBA10450;g__AV55;s__AV55 sp003219695	92.8%	3.4%	3.14	55.6%
GCA_003219115.1	f__UBA10450;g__AV69;s__AV69 sp003219115	82.4%	1.0%	2.67	55.3%
GCA_003219395.1	f__UBA10450;g__AV55;s__AV55 sp003219395	69.3%	2.2%	2.82	54.5%
GCA_003219415.1	f__UBA10450;g__AV55;s__AV55 sp003219415	97.8%	3.2%	3.62	54.4%

GBK assembly accession	GTDB Phylogeny	Compl.	Cont.	Size in Mbp	GC
GCA_003217875.1	f__UBA10450;g__AV55;s__AV55 sp003217875	86.8%	2.1%	2.45	54.6%
GCA_003220005.1	f__UBA10450;g__AV55;s__AV55 sp003220005	82.4%	0.7%	2.53	55.0%
GCA_002396485.1	f__*Terrimicrobiaceae*;g__UBA967;s__UBA967 sp002396485	98.7%	0.0%	2.77	56.2%
GCA_003217835.1	f__UBA10450;g__AV55;s__AV55 sp003217835	74.3%	0.7%	1.75	54.6%
GCA_003218525.1	f__UBA10450;g__AV69;s__AV69 sp003218525	78.0%	3.5%	2.43	55.3%
GCA_002452515.1	f__UBA6821;g__UBA6821;s__UBA6821 sp002452515	90.2%	1.4%	2.35	54.0%
GCA_003219945.1	f__UBA10450;g__AV55;s__AV55 sp003219945	81.5%	2.3%	2.24	54.9%
GCA_003218755.1	f__UBA10450;g__AV55;s__AV55 sp003218755	88.5%	3.2%	2.63	54.9%
GCA_003217895.1	f__UBA10450;g__AV55;s__AV55 sp003217895	94.3%	2.7%	2.74	55.0%
2651869889 (IMG Taxon ID)	not listed in the GTDB database (ncbi taxonomy: *Chthoniobacteraceae*; *Candidatus* Udaeobacter; *Candidatus* Udaeobacter copiosus)	80.0%	4.0%	2.66	54.3%
