# Supplementary material for: Globally Abundant “Candidatus Udaeobacter” Benefits from Release of Antibiotics in Soil and Potentially Performs Trace Gas Scavenging
Source: mSphere. 2020 Jul 8;5(4):e00186-20. doi: 10.1128/mSphere.00186-20 (PMC7343977; doi:10.1128/mSphere.00186-20)
Supplement: TABLE S5 [file mSphere.00186-20-st005.pdf]

Detection strategy	Contig/ scaffold	Annotation	Product	deepARG predicted resistance
deepARG	1	rlmN	Dual-specificity RNA methyltransferase RlmN	Phenicol
Prokka annotation	1	xerC_1	Tyrosine recombinase XerC	
Prokka annotation	3	xerC_2	Tyrosine recombinase XerC	
deepARG	5	Udaeo2_04600	multi-drug exprt ATP binding/permease protein	Multidrug
deepARG	6	phoP	Alkaline phosphatase synthesis transcriptional regulatory protein PhoP	Glycopeptide
Prokka annotation	6	ybhF_1	putative multidrug ABC transporter ATP-binding protein YbhF	
Prokka annotation	6	ybhR	putative multidrug ABC transporter permease YbhR	
Prokka annotation	7	Udaeo2_06040	Beta-lactamase	
deepARG	8	mdtA_1	multidrug resistance protein MdtA	multidrug
Prokka annotation	8	mdtC_1	Multidrug resistance protein MdtC	
Prokka annotation	9	fabI	Enoyl-[acyl-carrier-protein] reductase [NADH] FabI	
deepARG	11	macA_1	macrolide export protein	MLS
Prokka annotation	11	yknY_1	putative ABC transporter ATP-binding protein YknY	
Prokka annotation	11	macB_1	Macrolide export ATP-binding/permease protein MacB	
deepARG	11	rsmA	ribosomal RNA small subunit methyltransferase A	Aminoglycoside
deepARG	12	ybhF_2	multidrug ABC transporter ATP-binding protein	Bacitracin
deepARG	14	czcR	Transcriptional activator protein	Glycopeptide
Prokka annotation	14	macA_2	Macrolide export protein MacA	
deepARG	17	nreC	Oxygen regulatory protein NreC	Unclassified
deepARG	19	kdpD	sensor protein	Unclassified
Prokka annotation	21	mdtA_2	Multidrug resistance protein MdtA	
Prokka annotation	22	Udaeo2_12270	Beta-lactamase superfamily domain protein	
Diamond	23	Udaeo2_12390	IS5 family transposase IS1355	
Prokka annotation	27	Udaeo2_13820	IS110 family transposase ISGme8	
Prokka annotation	28	mdtA_3	Multidrug resistance protein MdtA	

Detection strategy	Contig/ scaffold	Annotation	Product	deepARG predicted resistance
deepARG	28	ybal	Putative cation/proton antiporterl YbaL	Fosmidomycin
Prokka annotation	30	vanB	Vancomycin B-type resistance protein VanB	
deepARG	40	spoVD	Stage V sporulation protein D	Beta-lactam
Diamond	42	xerD_1	Tyrosine recombinase XerD	
deepARG	46	bepE	Efflux pump membrane transporter	Multidrug
deepARG	46	srpA	Solvent efflux pump periplasmatic linker SrpA	Multidrug
Prokka annotation	49	Udaeo2_20030	IS110 family transposase ISGme8	
Diamond	49	Udaeo2_20040	IS3 family transposase ISStau1	
Diamond	61	xerD_2	Tyrosine recombinase XerD	
Prokka annotation	64	Udaeo2_23300	Beta-lactamase	
Prokka annotation	69	macB_2	Macrolide export ATP-binding/permease protein MacB	
Prokka annotation	69	macB_3	Macrolide export ATP-binding/permease protein MacB	
deepARG	69	Udaeo2_24310	Putative multidrug export ATP-binding/permease protein	Multidrug
Prokka annotation	74	mdtA_4	Multidrug resistance protein MdtA	
Prokka annotation	74	mdtB_1	Multidrug resistance protein MdtB	
Prokka annotation	74	mdtB_2	Multidrug resistance protein MdtB	
Prokka annotation	74	mdtC_2	Multidrug resistance protein MdtC	
Prokka annotation	78	xerC_3	Tyrosine recombinase XerC	
Prokka annotation	80	xerC_4	Tyrosine recombinase XerC	
Prokka annotation	89	lnrL	Linearmycin resistance ATP-binding protein	
Prokka annotation	89	lnrN	Linearmycin resistance permease protein LnrN	
deepARG	89	syrM1	HTH-type transcriptional regulator SyrM 1	
deepARG	92	Udaeo2_28300	Putative multidrug export ATP-binding/permease protein	Multidrug
Diamond	94	recA	Protein RecA	
deepARG	98	tcrA	Transcriptional regulatory protein	Glycopeptide

Detection strategy	Contig/ scaffold	Annotation	Product	deepARG predicted resistance
Prokka annotation	99	Udaeo2_29330	macB: subunit of efflux pump conferring antibiotic resistance	
Prokka annotation	99	Udaeo2_29350	macB-like periplasmic core domain protein	
Prokka annotation	99	yknY_4	putative ABC transporter ATP-binding protein YknY	
Prokka annotation	99	Udaeo2_29370	HlyD family secretion protein	
Prokka annotation	106	mdtA_5	Multidrug resistance protein MdtA	
Prokka annotation	106	mdtC_3	Multidrug resistance protein MdtC	
Prokka annotation	109	Udaeo2_30640	Metallo-beta-lactamase superfamily protein	
Prokka annotation	110	macB_4	Macrolide export ATP-binding/permease protein MacB	
Prokka annotation	110	Udaeo2_30740	MacB-like periplasmatic core domain protein	
Prokka annotation	110	Udaeo2_30700	MacB-like periplasmatic core domain protein	
Prokka annotation	110	Udaeo2_30690	MacB-like periplasmatic core domain protein	
Prokka annotation	110	Udaeo2_30660	macB: subunit of efflux pump conferring antibiotic resistance	
Prokka annotation	110	Udaeo2_30670	macB: subunit of efflux pump conferring antibiotic resistance	
deepARG	112	stp	Multidrug resistance protein Stp	Tetracenomycin_C
deepARG	116	blaP	Beta-lactamase	Beta-lactam
deepARG	125	Udaeo2_32650	Metallo-beta-lactamase superfamily protein	Beta-lactam
Prokka annotation	131	Udaeo2_33250	Transposase IS116/IS110/IS902 family protein	
Diamond	132	xerC_5	Tyrosine recombinase XerC	
